# Multidimensional analysis of deubiquitinating enzymes in colorectal cancer: biological mechanisms and targeted therapeutic strategies

**DOI:** 10.3389/fonc.2026.1808932

**Published:** 2026-04-23

**Authors:** Ting Wang, Pingying Li, Shuo Xu, Guocai Xu

**Affiliations:** 1 Qinghai University, Xining, China; 2 Qinghai Provincial People's Hospital, Xining, China

**Keywords:** colorectal cancer, deubiquitinating enzymes, immune evasion, metabolic reprogramming, non-apoptotic cell death, proteolytic-targeted chimeras

## Abstract

Colorectal cancer (CRC) is one of the most common and deadly cancers worldwide. Its significant heterogeneity and cellular plasticity are key drivers of clinical treatment challenges and drug resistance. Deubiquitinating enzymes (DUBs) play a critical role in the mechanisms regulating the onset and progression of CRC. As key functional molecules of the ubiquitin-proteasome system (UPS), DUBs exert central influences on processes such as DNA damage response and repair, metabolic reprogramming, non-apoptotic cell death, and clinical treatment outcomes in CRC cells. This article systematically elucidates the key molecular mechanisms of DUBs in CRC from multiple perspectives, with a particular focus on their bridging role between intrinsic stress adaptation and tumor immune evasion in cancer cells. Furthermore, this article critically evaluates the evolution of DUB-targeting strategies, from the limitations of traditional small-molecule inhibitors to technological innovations such as protein degradation-targeting chimeric proteins (PROTACs) and deubiquitinase-targeting chimeric proteins (DUBTACs). These strategies aim to reverse multidrug resistance by degrading oncogenic DUBs or stabilizing tumor-suppressor proteins, thereby providing new research leads and potential translational directions for precision therapy in CRC.

## 1 Introduction

Colorectal cancer (CRC) is one of the most common and deadly cancers worldwide (1, 2). Despite continuous advances in screening, surgery, chemotherapy, targeted therapy, and immunotherapy, the prognosis for patients with advanced CRC—particularly those with distant metastases—remains poor, with a 5-year survival rate of approximately 15% (1). Therefore, further elucidation of the molecular mechanisms underlying CRC development and identification of potential therapeutic targets remain critical in this field (1). Within the ubiquitin-proteasome system (UPS), which maintains cellular protein homeostasis, deubiquitinating enzymes (DUBs) remove ubiquitin modifications from substrate proteins, thereby regulating protein stability, subcellular localization, activity, and protein interactions (2, 3). Currently, DUBs are generally classified into seven families: ubiquitin-specific proteases (USP), ubiquitin C-terminal hydrolases (UCH), ovarian tumor domain proteases (OTU), Machado-Joseph domain proteases (MJD), JAB1/MPN/MOV34 metalloproteases (JAMM), the MINDY family, and the ZUFSP family (2, 3). Previous studies have suggested that various DUBs exhibit abnormal expression or dysfunction in CRC and may contribute to tumor proliferation, invasion, metastasis, immune evasion, and treatment resistance by influencing pathways such as NF-κB, PI3K/Akt, p53, and Wnt/β-catenin (2–4). Based on this, this article focuses on DUBs in CRC for which direct functional evidence exists and that are closely associated with APC/Wnt dysregulation, DNA damage repair, immune evasion, metabolic reprogramming, and treatment resistance, such as USP1, USP7, USP10, USP14, USP22, and UCHL1 (2–4). The full text summarizes these findings across five aspects: malignant phenotypes of tumor cells, metabolic reprogramming, non-apoptotic cell death, the tumor immune microenvironment (TIME), and clinical translation. It further discusses the boundaries between CRC-specific mechanisms and pan-cancer shared mechanisms, as well as the practical challenges and future directions for DUB-targeted therapies (2–4).

### 1.1 A functional convergence framework for the DUB family–substrate–signal pathway in CRC

From a family classification perspective, although CRC-related DUB research involves multiple families such as USP, UCH, OTU, JAMM, MJD, MINDY, and ZUFSP, the functions of these molecules are not isolated or unrelated to one another (2, 3). Existing studies indicate that members of different families ultimately converge on several common CRC progression pathways, including Wnt/β-catenin and stemness maintenance, DNA damage repair and chemoresistance, metabolic reprogramming, non-apoptotic cell death thresholds, and tumor immune evasion. This phenomenon suggests that the role of DUBs in CRC is not simply a matter of “each regulating a single substrate,” but rather that they collectively shape the malignant phenotype of CRC by modulating a small number of high-level biological processes that determine tumor progression and treatment response (2–4).

From a mechanistic perspective, this “functional convergence” does not imply that different DUBs are interchangeable, nor does it mean that their functions are identical. A more reasonable interpretation is that the core thresholds truly determining the biological behavior and therapeutic outcomes of CRC are concentrated at a few key levels, such as APC/Wnt-driven stemness and cellular plasticity, replicative stress and DNA damage response, metabolic adaptation, sensitivity to non-apoptotic cell death, and reduced tumor immunogenicity (2–4). Although DUBs differ in catalytic structure, substrate profiles, and cellular localization, they may ultimately contribute to the onset, progression, and treatment resistance of CRC by influencing these critical thresholds. Therefore, rather than viewing DUBs as a group of isolated deubiquitinating enzymes, it is more appropriate to regard them as regulatory nodes of protein homeostasis that connect different phenotypic levels (2–4).

This framework is particularly important for understanding the research value of DUBs in CRC. First, it helps explain why DUBs from different families ultimately recur in a few major pathways, such as Wnt/β-catenin, DNA damage repair, metabolism, and immune evasion; second, it suggests that future research should not merely focus on continuously expanding the list of DUBs, but should further identify which DUBs truly occupy the intersection of multiple key phenotypic axes, and which are more likely to function as local regulatory molecules within specific pathways. In other words, the focus of DUB research in CRC should not be limited to “discovering more relevant molecules.” However, it should gradually establish the correspondence between “core functional nodes—applicable contexts—potential translational value”.

**Figure 1:**
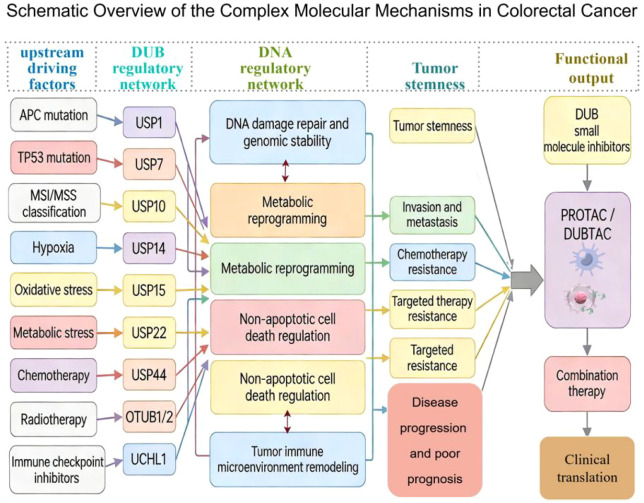
A simplified conceptual framework of DUB families—substrates—pathways—phenotypes (2–4). The left side shows different DUB families; the center summarizes representative substrates and pathways involved in CRC; and the right side corresponds to major phenotypes such as Wnt signaling/stemness maintenance, DNA damage repair/chemotherapy resistance, metabolic reprogramming, non-apoptotic cell death, and immune evasion (2–4). The primary purpose of this figure is not to exhaustively list all reported DUBs, but rather to highlight that although members of different families originate from diverse sources, they often functionally converge in CRC on a few key biological levels that determine tumor progression and treatment response. This figure is primarily intended to help readers quickly grasp the overall logical structure of the full text and to provide a framework for the subsequent discussion, which is organized according to three dimensions: functional mechanisms, evidence grading, and translational significance (2–4) (Created by Figdraw).

Based on this understanding, this article, building upon a chapter-by-chapter review, further adopts an analytical framework of “DUB family—key substrate—signaling pathway—CRC phenotype—translational significance” to help readers grasp the common points of action and differences among various DUBs in CRC from a holistic perspective, and to distinguish as much as possible which mechanisms already have relatively direct evidence in CRC and which still primarily belong to pan-cancer references or inferential directions (2–4) (**Figure 1**). From the perspective of this paper, the significance of this framework lies not only in facilitating the synthesis of review writing but also in providing a clearer logical foundation for subsequent tiered research and targeted translation. What truly warrants priority attention is often not the most frequently reported DUBs, but rather those nodes that can simultaneously link multiple key phenotypic processes and demonstrate intervenability in specific CRC contexts.

## 2 Literature search and evidence grading

To ensure the systematic, objective, and reproducible nature of this review, this study followed established procedures for literature search and screening.

### 2.1 Literature search strategy

This study searched the following two biomedical literature databases:

- PubMed (https://pubmed.ncbi.nlm.nih.gov/).
- Web of Science (https://www.webofscience.com/).

The search time frame was from January 1, 2000, to March 26, 2026. A two-stage search strategy was employed: an initial systematic search was conducted through October 16, 2025; a supplementary search was then conducted focusing on CRC-specific mechanisms, evidence grading, clinical translation, and entries related to **Table 1**, with the final update completed on March 26, 2026.

**Table 1:** Table of translational resources for DUB/UPS drug targets related to colorectal cancer (including substrates/pathways, CRC model context, and strength of evidence).

Target	Representative molecule	CRC-related substrates or pathways	Background of the CRC model	Levels of evidence	Development phase	Evidential attributes and key conversion issues
USP7(DUB) (4–8)	P5091;FT671	β-catenin/Wnt (APC background); mutant p53; potential for combination therapy with immune checkpoint inhibitors and anti-PD-1 in MSS CRC	CRC cell lines; APC-related models/organoids; patient samples	Level 1-2	Preclinical	CRC is context-dependent; it is influenced by factors such as p53 genotype, APC background, drug selectivity, and USP22 compensation
USP1(DUB) (9)	ML323	FANCD2;PCNA;DDR	CRC cell line	Level 3	Preclinical	There is already direct evidence in CRC, but it pertains to a pan-cancer shared mechanism; it is more suitable for use in combination with PARP inhibitors or platinum-based agents, and attention must be paid to toxicity in normal tissues
USP1(DUB) (9, 10)	KSQ-4279(RO7623066)	In conjunction with the USP1-DNA repair axis	Focuses primarily on pan-cancer clinical development; limited direct evidence for CRC	Level 4	Phase I clinical trial	Currently, the evidence is primarily based on pan-cancer clinical trials; efficacy in CRC has not yet been established and requires stratification based on biomarkers
UBA1/E1 (Not DUB; comparator drug for UPS) (11)	TAK-243(MLN7243)	Ubiquitin activation pathway	Not specific to CRC; focuses primarily on early-stage research on hematologic malignancies and solid tumors	Level 4	Phase I clinical trial	Cannot be classified as a USP1 or DUB inhibitor; serves only as a comparator drug for UPS, with limited evidence in CRC
USP14(DUB) (4, 12, 13)	IU1 Series	JNK/ Mitogen-Activated Protein Kinase (MAPK)	CRC cell lines + *in vivo* models	Level 2	Preclinical	There is already direct evidence in CRC, but it is more likely to represent a pan-cancer shared mechanism; attention should be paid to proteasome-related systemic toxicity, and catalytic inhibition may not fully mimic gene deletion
UCHL1(DUB) (4, 14)	LDN-57444	Promotes the β-catenin pathway and maintains tumor stemness	CRC: *In vitro* + *in vivo* (early evidence)	Level 2	Preclinical	While is already some early direct evidence in CRC, further validation is needed in modern models such as organoids and patient-derived xenografts (PDX); the therapeutic window is limited by high expression in brain tissue

The search strategy combined subject headings with free-text terms. DUB-related search terms included: “deubiquitinating enzyme,” “deubiquitinase,” “DUB,” “USP,” “UCH,” “OTU,” “MJD,” “JAMM,” “MINDY,” and “ZUFSP”; CRC-related search terms included: “colorectal cancer,” “CRC,” “colon cancer,” and “rectal cancer.” During the supplementary search phase, combined searches were also conducted for keywords mentioned in the peer review comments, such as “CRC-specific,” “pan-cancer,” “USP7 APC mutation,” “USP1 inhibitor,” “TAK-243 UBA1,” “PROTAC,” “DUBTAC,” “organoid,” “Patient-Derived Xenograft (PDX),” and “patient-derived”.

### 2.2 Inclusion and exclusion criteria

Inclusion Criteria: (1) Research Content: Studies addressing the expression, function, regulatory mechanisms, or therapeutic potential of DUBs, with direct or indirect relevance to CRC; (2) Study Type: Studies involving cytological experiments (*in vitro*), organoid models, animal models (*in vivo*), or patient sample data; or pan-cancer studies providing significant insights into underlying mechanisms; (3) Language: Published in English; (4) Publication Type: Original research or reviews (reviews are accepted only for background supplementation and mechanism clarification).

Exclusion Criteria: (1) Research content completely unrelated to DUB mechanisms; (2) Studies reporting only gene expression data without providing any functional validation or mechanistic analysis (though such studies may be cited as background); (3) Studies presented as conference abstracts, preprints (not formally published), or retracted publications; (4) Studies with serious methodological flaws or conclusions that have been definitively refuted by subsequent research.

Special Notes: Given that research on DUBs in CRC is still in a rapidly evolving stage, some pioneering discoveries regarding regulatory mechanisms (such as copper-mediated cell death, pyroptosis, and the regulation of related transporters) originate from other cancer types or basic research. For such studies, if the insights into the mechanisms provide significant reference value for CRC, this review will include them, with a clear annotation in the main text stating “This mechanism requires further validation in CRC” or “Primarily based on evidence from other cancer types”.

### 2.3 Grading of evidence strength

To objectively assess the strength of evidence supporting the conclusions of each study, this study adopts the following four-tier grading system. The corresponding level of evidence will be indicated at key conclusions in the main text:

Level 1: CRC patient samples (tissue or blood) + *in vivo* animal models + validation of molecular mechanisms (e.g., deubiquitination experiments, site-specific mutations, etc.); experimental model types: patient cohorts + *in vivo* + *in vitro*.
Level 2: *In vivo* animal models of CRC + validation of molecular mechanisms (lacking validation with patient samples); experimental model types: *in vivo* + *in vitro*.
Level 3: CRC cell lines + validation of molecular mechanisms (lacking *in vivo* or patient sample validation); experimental model type: *in vitro*.
Level 4: Evidence from non-CRC cancer types (e.g., lung cancer, breast cancer, etc.), or studies reporting only correlation analyses (without validation of mechanisms); experimental model type: pan-cancer/correlation.

### 2.4 Handling of conflicting literature

When conflicting conclusions exist regarding the same scientific question, this review prioritizes the following principles: (1) studies with a higher level of evidence; (2) studies published more recently; (3) studies with larger sample sizes and more rigorous experimental designs. If necessary, conflicting conclusions will be objectively presented in the main text, along with a discussion of possible reasons (e.g., differences in cellular background, experimental conditions, or tumor subtypes).

## 3 The role of DUBs in shaping the malignant characteristics of CRC cells

### 3.1 Mechanisms of DUB-mediated DNA damage repair and chemotherapy sensitization

DUBs participate in DNA damage recognition, repair complex assembly, and checkpoint signaling regulation by deubiquitinating mono- or polyubiquitin chains on substrate proteins (2, 4). This process is particularly important for CRC, as oxaliplatin, 5-FU, and radiotherapy all induce varying degrees of replicative stress and DNA damage responses, thereby influencing treatment sensitivity and the development of acquired resistance (1, 4).

In *TP53*-wild-type CRC cells, the stabilizing effect of USP7 on MDM2 and MDMX is typically more pronounced; consequently, its overall effect often manifests as an indirect reduction in p53 levels (15–18) (Evidence Level: Level 3; Model Type: *In vitro* CRC cell lines). Under DNA damage stress, signaling pathways such as ATM can alter USP7’s binding preferences, shifting its interaction from MDM2/MDMX to p53, thereby enhancing p53-mediated cell cycle arrest or apoptosis (15–18). In contrast, in *TP53*-mutant CRC, USP7 is more likely to stabilize mutant p53 directly. A 2024 study on CRC demonstrated that USP7 protein levels are elevated in *TP53*-mutant CRC cells and cancer stem cell-enriched populations; both *USP7* knockdown and treatment with P5091 reduce mutant p53 protein levels and inhibit self-renewal capacity (5) (Evidence Level: Level 3; Model Type: *In vitro* + Mechanism Validation, supported by partial patient-related evidence). Therefore, USP7 should not be simplistically characterized as a single-pathway target that merely “stabilizes p53” or “restores p53 function.” A more appropriate description is that the effects of USP7 on the p53 axis are significantly genotype- and substrate-competition-dependent, and its therapeutic value requires stratified evaluation based on *TP53* status, *APC* background, and relevant substrate networks (5, 6, 15–19). Similarly, DDR-associated DUBs such as USP1 may enhance CRC’s response to DNA damage therapy, but these remain primarily mechanism-based preclinical inferences and should not be directly extrapolated to all CRC patients (9).

In addition to USP1 and USP7, direct evidence from CRC studies over the past two years has expanded the spectrum of DDR-associated DUBs (20–24). A 2025 study demonstrated that USP10 binds to XAB2, removes its K48-linked polyubiquitin chains, and stabilizes XAB2, with a relatively clear molecular basis at the K593 site; the stabilized XAB2 further upregulates ANXA2 transcription, enhances DNA damage repair, and promotes oxaliplatin resistance (20). In contrast, while there is evidence that USP28 promotes c-MYC stability and glycolysis in CRC, most evidence supporting its role as a “DDR/therapeutic resistance” molecule still comes from other cancer types and mechanistic studies; therefore, it is currently more appropriate to regard it as an emerging DUB candidate in CRC (21, 22). OTUD5 has been associated with genomic stability, p53 regulation, and telomere-related DNA repair in pan-cancer studies; however, direct perturbation evidence of the same caliber is still lacking in CRC. Therefore, this paper retains it as a direction worthy of attention rather than an established conclusion (23, 24).

Based on the above studies, research on DDR-related DUBs in CRC is gradually shifting from an understanding of “individual repair protein stability” to that of “replication stress adaptation networks.” In other words, what truly warrants priority attention is not merely whether a specific substrate is deubiquitinated, but rather who determines the therapeutic thresholds for CRC in response to platinum agents, 5-FU, or radiotherapy. Based on current evidence, USP1 is closer to the “synergistic sensitization” node, USP7 is closer to the “background-dependent” node, while USP10 suggests the potential existence of a more direct, emerging DDR-DUB axis in chemotherapy-acquired resistance. In contrast, USP28 and OTUD5 are currently better suited as avenues for mechanistic exploration rather than mature CRC targets with clear translational priorities.

### 3.2 Key regulatory roles of DUBs in histone modification and epigenetic processes

**Figure 2:**
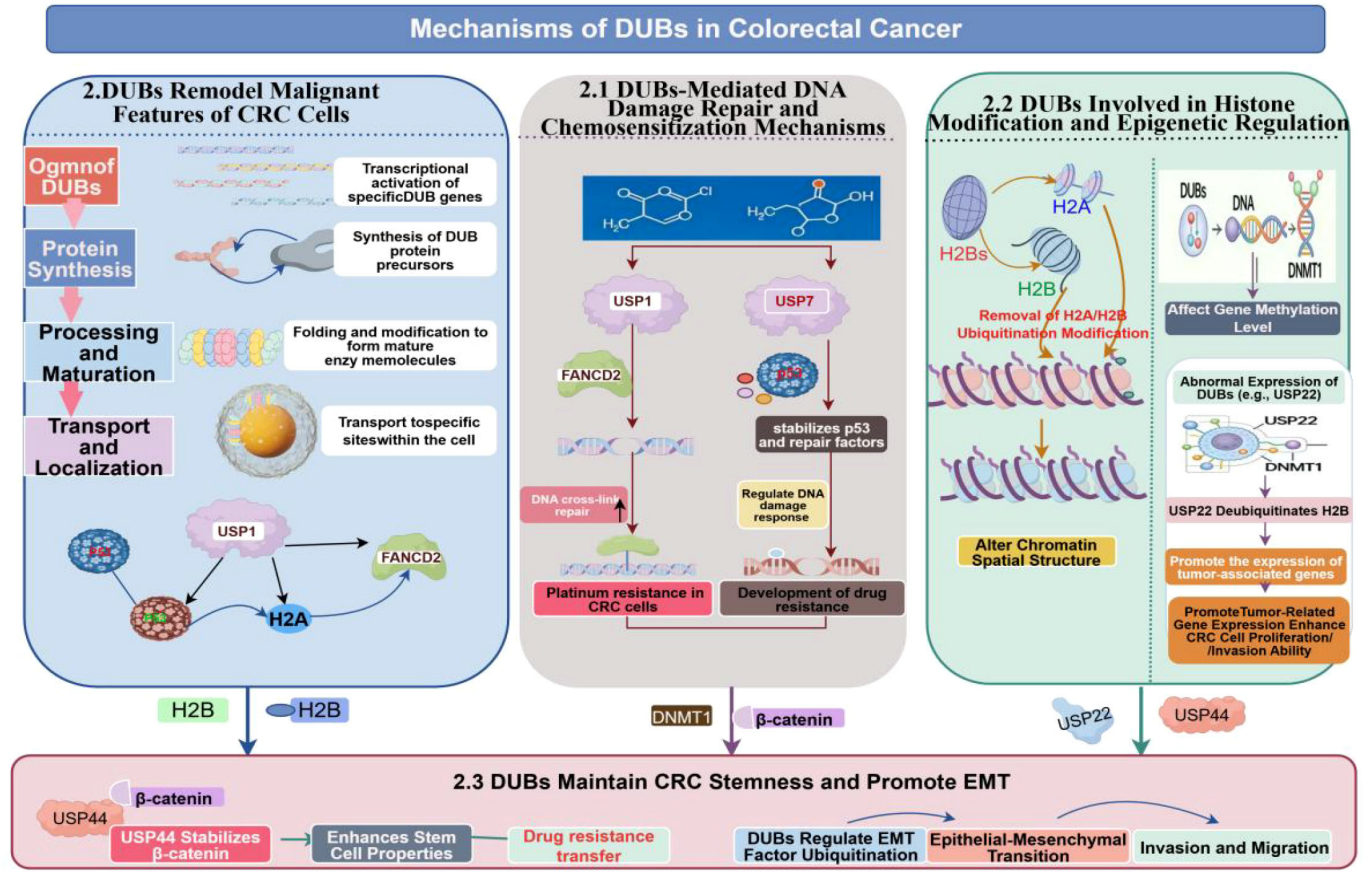
This figure summarizes the three main mechanisms by which DUBs regulate tumor malignancy in CRC (2–4). It aims to outline the three primary mechanisms through which DUBs influence the malignant phenotype in CRC, including DNA damage repair and treatment resistance, epigenetic regulation, and stemness/epithelial-mesenchymal transition (EMT)-related plasticity remodeling. Solid lines indicate associations supported by relatively direct evidence in CRC, while dashed lines indicate relationships inferred primarily from other cancer types or basic research that require further validation in CRC (2–4). Representative pathways with relatively direct evidence in CRC include: the USP1–FANCD2-related DNA cross-link repair pathway, suggesting that DUBs can influence sensitivity to platinum-based therapy; the USP7–p53 axis, whose function is highly context-dependent: in the TP53 wild-type context, it primarily inhibits p53 indirectly by stabilizing MDM2/MDMX, whereas in the TP53 mutant context, it is more likely to stabilize mutant p53 and promote tumor progression directly; the USP22–H2B deubiquitination axis, suggesting that DUBs may participate in the remodeling of tumor-associated transcriptional programs; and the USP44–Axin1–Wnt/β-catenin axis, suggesting that not all Wnt-associated DUBs act in a pro-tumorigenic manner, as some DUBs may also exhibit context-dependent tumor-suppressive regulation (5, 6, 9, 25–29). The association between DNMT1 stability and DNA methylation depicted in the figure is currently based primarily on evidence from non-CRC sources; therefore, it is retained here only as a speculative direction (25). The focus of this figure is not to exhaustively list all reported DUBs, but rather to emphasize that although members of different families originate from diverse sources, they often functionally converge in CRC on a few key biological levels that determine tumor progression and treatment response; simultaneously, the maturity of evidence for these mechanisms varies, and they cannot be understood as being on the same level (Created by Figdraw).

DUBs also play a significant role in epigenetic regulation (**Figure 2**); they can alter chromatin states by removing ubiquitin modifications from histones H2A and H2B, thereby influencing transcriptional programs (25). Regarding current direct evidence in CRC, USP22 is the most extensively studied epigenetically relevant DUB (26, 27). Multiple studies suggest that high USP22 expression is associated with CRC invasion, metastasis, and poor prognosis, and that it participates in tumor-related transcriptional programs through H2B deubiquitination; However, other studies have shown that USP22 may exhibit context-dependent or even tumor-suppressive effects in certain CRC contexts. For example, Kosinsky et al. found in *Apc*-mutant mice and human CRC models that USP22 deficiency enhances mTOR activity and increases tumor burden, suggesting a context-dependent tumor-suppressive role for USP22 in certain CRC contexts (26, 27).

Therefore, a more appropriate characterization of USP22 is not to classify it as a “pure oncogene,” but rather to emphasize that its function exhibits significant context-dependence. In other words, USP22 in CRC is not a linear molecule with a constant direction across all models, but rather functions more as an epigenetic regulatory node jointly influenced by molecular subtypes, chromatin states, and downstream transcriptional networks (26, 27). This suggests that when DUBs are involved in chromatin and transcriptional regulation, classifying them as oncogenic or tumor-suppressive based solely on elevated expression or phenotypes in a single model is often insufficient to reflect their true function.

Based on current evidence, the most significant aspect of epigenetically associated DUBs in CRC is not the identification of additional new histone deubiquitinating molecules, but rather determining whether the direction of these molecules’ actions can be predicted by molecular context. Regarding USP22, the most valuable questions for future research are: Does its function depend on the APC/Wnt context, mTOR activity status, or specific transcriptional programs? Only after these questions are further resolved can USP22 evolve from a frequently discussed epigenetic DUB into a functional node in CRC that can be utilized in a stratified manner.

### 3.3 DUBs as key molecules in CRC stemness maintenance and epithelial-mesenchymal transition

DUBs also play a crucial role in the self-renewal of colorectal cancer stem cells (CSCs) and the epithelial-mesenchymal transition (EMT) process (2, 30). Studies have shown that certain DUBs can influence CRC stemness maintenance, cellular plasticity, and metastatic potential by regulating pathways such as Wnt/β-catenin and Notch (2, 30). However, the roles of different DUBs in this process are not (2). For example, USP44 does not stabilize β-catenin and promote stemness in CRC; On the contrary, existing CRC studies suggest that USP44 stabilizes Axin1 through deubiquitination, thereby inhibiting Wnt/β-catenin signaling and exerting an anticancer effect (28) (Evidence Level: Level 2; Model Type: *In vitro* + *In vivo*). Therefore, USP44 is more appropriately described as a “Wnt-inhibitory DUB in CRC” rather than a pro-cancerous stemness-maintaining factor (28). This finding itself suggests that when DUBs participate in the regulation of stemness and EMT, their effects do not necessarily follow a single direction of “enhancing plasticity and invasiveness,” but rather are highly dependent on the specific signaling pathway context and substrate properties.

On the other hand, other DUBs may promote the transition of cells from an epithelial phenotype to a mesenchymal phenotype by influencing the stability and transcriptional programs of EMT-related factors, thereby enhancing invasiveness and migratory capacity (19, 30). Based on the existing literature, DUBs in this field are not merely linked to the “EMT phenotype” itself, but rather to the plasticity threshold of CRC cells: that is, the conditions under which tumor cells maintain a stem-like state, acquire migratory capacity, and develop resistance to treatment. Precisely for this reason, DUB networks should not be understood as unidirectional oncogenic modules in stemness/EMT-related research, but rather as proteostatic networks that regulate the stability of cellular fate and the capacity for phenotypic transition.

Therefore, the top priority in this field is not to continue expanding the list of Wnt- or EMT-associated DUBs, but to clarify which DUBs truly determine the maintenance thresholds of CRC cell plasticity and which are merely co-regulators of this phenotype. This distinction is particularly critical for subsequent translation, as only those DUBs situated at the intersection of “stemness maintenance—phenotypic transition—therapeutic resistance” are more likely to become candidate targets with practical intervention value.

Summary of this section: Overall, the reshaping of the malignant phenotype of CRC by DUBs is primarily concentrated at three levels: DNA damage repair and treatment resistance, epigenetic regulation, and stemness/EMT-related cellular plasticity. However, these effects are not a simple superposition of several independent molecular axes, but rather resemble a multilayer regulatory network governing treatment thresholds, chromatin states, and the capacity for phenotypic conversion (2–4). Based on current direct evidence in CRC, USP1, USP7, USP22, and USP44 represent relatively more established pathways: The first two are closer to key nodes in DNA damage response and treatment sensitivity; USP22 suggests that epigenetically associated DUBs exhibit significant context-dependence, while USP44 indicates that not all Wnt-associated DUBs act in a pro-oncogenic manner (5, 6, 9, 20–24, 26–28). In contrast, USP10, USP28, and OTUD5 are better suited as emerging nodes worthy of further investigation; they suggest that the DUB network in CRC is still evolving from “single-substrate regulation” toward a higher-level understanding of adaptation to replicative stress, chromatin remodeling, and thresholds of cellular plasticity (20–24). Therefore, the priority for future research should not be to continue expanding the list of DUBs, but rather to identify which DUBs truly determine the critical thresholds for CRC’s response to DNA damage, transcriptional remodeling, and phenotypic conversion, and to establish a clearer hierarchical and translational framework based on these findings.

## 4 Regulatory mechanisms of DUBs in metabolic reprogramming of CRC

### 4.1 Regulatory mechanisms of DUBs at key nodes of glycolysis in CRC

**Figure 3:**
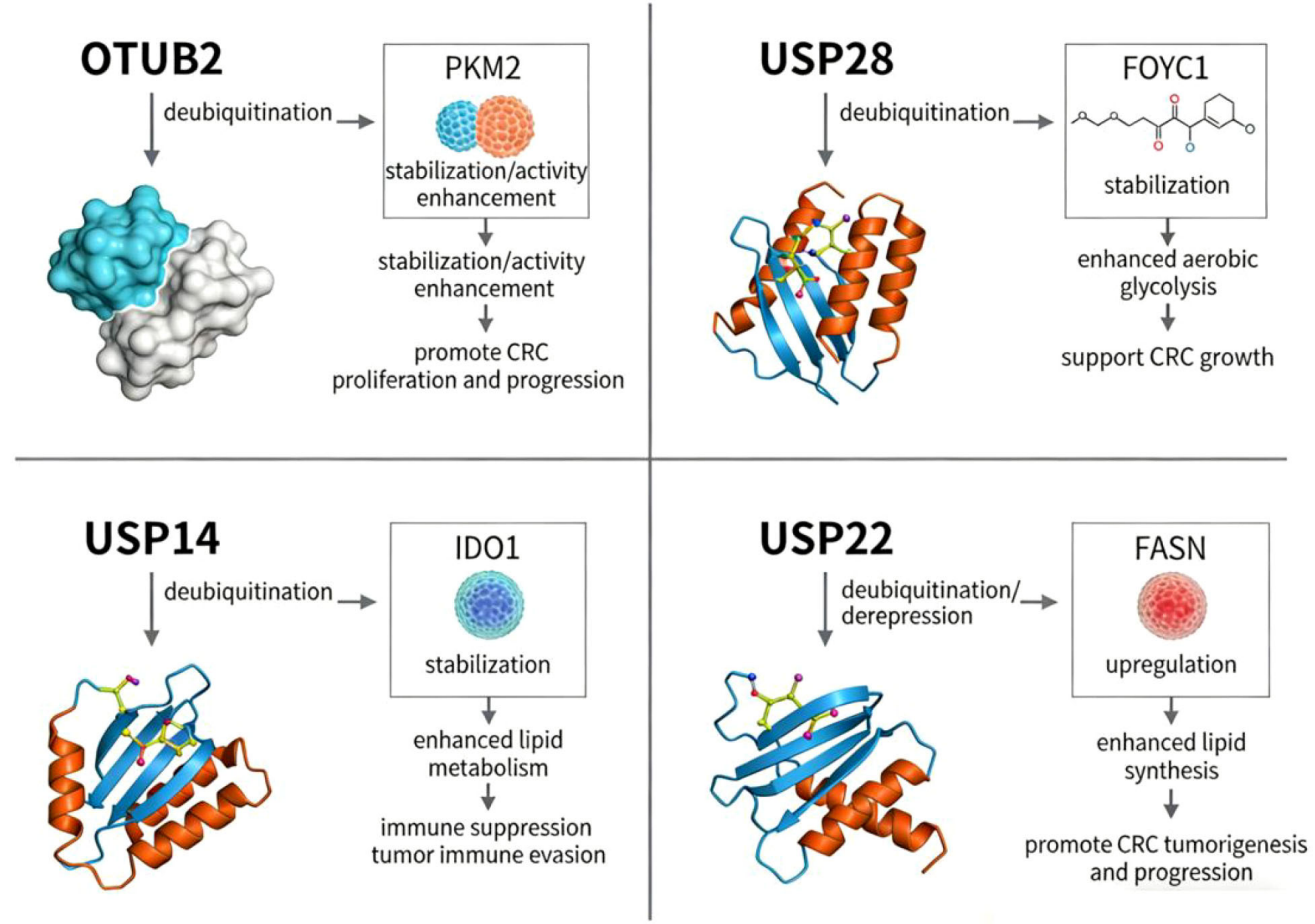
This figure illustrates representative mechanisms by which DUBs participate in metabolic reprogramming in CRC (12, 31, 32). The figure highlights only the four metabolism-related DUB axes for which evidence in CRC is currently relatively direct, including the OTUB2–PKM2 glycolysis axis, the USP28–FOXC1 glycolysis axis, the USP14–IDO1 tryptophan metabolism axis, and the USP22–FASN lipid synthesis axis. Solid lines indicate associations supported by direct studies in CRC (12, 22, 31, 32). The remaining metabolic associations involving GLUT1, CD36, GLS1, and others are currently based primarily on findings from other cancer types or basic research and lack sufficient direct validation in CRC; therefore, they are not included in this figure (4). This figure aims to highlight that the maturity of evidence across different metabolic axes is inconsistent (Created by Figdraw).

In recent years, metabolic reprogramming—particularly enhanced glycolysis—has been recognized as one of the key mechanisms underlying the development and progression of CRC (2, 4). DUBs can influence the stability and function of key metabolic enzymes by regulating their ubiquitination levels (2, 4). In CRC, the most direct evidence in this regard comes primarily from OTUB2 rather than USP29. Original studies have shown that OTUB2 can enhance PKM2 activity through deubiquitination and promote glycolysis, thereby accelerating CRC progression (31) (**Figure 3**) (Evidence Level: Level 3; Model Type: *In vitro* CRC cell lines). In contrast, the USP7/USP10-GLUT1 axis and several FASN-related DUB axes are currently inferred to apply to CRC primarily based on other cancer types or basic research; This should be clearly marked as pending verification in the CRC. This suggests that DUBs may indeed participate in metabolic regulation in CRC, but the maturity of evidence varies significantly across different metabolic axes, and it is inappropriate to treat them uniformly as established conclusions (4).

At the molecular level, the role of OTUB2 in PKM2 is not merely a general “stabilization,” but rather involves inhibiting the interaction between PKM2 and the E3 ligase Parkin, thereby reducing PKM2 ubiquitination and enhancing its enzymatic activity, which helps CRC cells maintain metabolic adaptation under glucose-deprivation stress (31). On the other hand, USP28 enhances aerobic glycolysis in CRC by increasing FOXC1 stability, suggesting that metabolism-related DUBs not only influence substrate abundance but may also participate in transcriptional-metabolic coupling under stress conditions (22). However, the maturity of evidence in this field remains uneven: while the glycolytic axis has relatively direct evidence in CRC, DUBs associated with lipid and glutamine metabolism largely remain at the stage of mechanistically plausible but requiring validation in CRC (12, 22, 32).

### 4.2 Mechanisms by which DUBs regulate lipid metabolism in CRC cells via fatty acid synthesis and uptake

Lipid metabolic reprogramming is a critical foundation for CRC to maintain its growth and invasive capabilities; however, direct evidence linking this to DUBs is currently limited. In CRC, the most well-defined DUB axis related to lipid metabolism is primarily centered on the USP22-FASN axis (32). Previous studies have shown that under conditions of p53 deficiency and oxidative stress, the USP22-FASN axis promotes lipid synthesis and drives tumorigenesis. In contrast, other DUB mechanisms involving fatty acid uptake or the utilization of exogenous lipids are currently based primarily on inferences from other cancer types or basic research and lack sufficient direct validation in CRC (4). Therefore, at this stage, it is more appropriate to summarize the relationship between DUBs and lipid metabolism in CRC as having preliminary direct evidence, but the underlying mechanisms still require further exploration.

### 4.3 DUB-mediated reprogramming of amino acid metabolism

Amino acid metabolism plays a role in energy supply, biosynthesis, and the maintenance of redox homeostasis in CRC cells (4). Among these, both glutamine and tryptophan metabolism are associated with tumor growth and treatment response. Current research indicates that enhanced glutamine metabolism in CRC may be mediated by key enzymes such as GLS1 and GPT2 and is associated with invasion, metastasis, and molecular subtyping; however, regarding DUBs, the most direct evidence in CRC currently focuses primarily on tryptophan metabolism rather than glutamine catabolism (4). Previous studies have shown that USP14 can stabilize IDO1 through deubiquitination, thereby promoting tryptophan metabolism and enhancing immunosuppression (12). In contrast, there remains a lack of sufficient direct experimental evidence to support whether DUBs directly participate in CRC metabolic reprogramming by regulating GLS1 or other glutamine metabolic enzymes (4). Furthermore, aside from tryptophan metabolism, whether DUBs directly regulate other amino acid metabolism-related enzymes and drive CRC metabolic reprogramming requires further direct evidence (4) (**Figure 3**).

Summary of this section: Overall, DUB-mediated metabolic reprogramming has become a key entry point for understanding CRC growth adaptation, immune suppression, and treatment resistance; however, there are significant differences in the strength of evidence across different metabolic pathways. Based on current direct evidence in CRC, mechanisms related to glycolysis are relatively the most well-established. Among these, the OTUB2–PKM2 and USP28–FOXC1 axes suggest that DUBs not only enhance aerobic glycolysis by stabilizing key metabolic enzymes or transcription factors but may also participate in the adaptive remodeling of tumor cells under metabolic stress conditions. Regarding amino acid metabolism, the USP14–IDO1 axis further suggests that DUB-mediated metabolic regulation not only serves tumor proliferation but may also contribute to the formation of an immunosuppressive microenvironment through tryptophan metabolism. In contrast, direct evidence regarding lipid metabolism is currently relatively clear for the USP22–FASN axis, whereas DUB mechanisms involving fatty acid uptake, GLUT1-related regulation, or glutamine catabolism remain primarily at the stage of inference or indirect support in other cancer types. Thus, while a link between DUBs and metabolic reprogramming in CRC has been established, the molecular landscape remains incomplete. Moving forward, the priority should not be to continue expanding the list of metabolism-related DUBs in parallel, but rather to further identify the key nodes that truly determine the metabolic vulnerability of CRC, focusing on glycolysis, tryptophan metabolism, and their connections to treatment resistance or immune suppression.

## 5 The key role of DUBs in non-apoptotic cell death in CRC

### 5.1 Validated mechanisms and hypothesized pathways of DUBs in CRC ferroptosis

**Figure 4:**
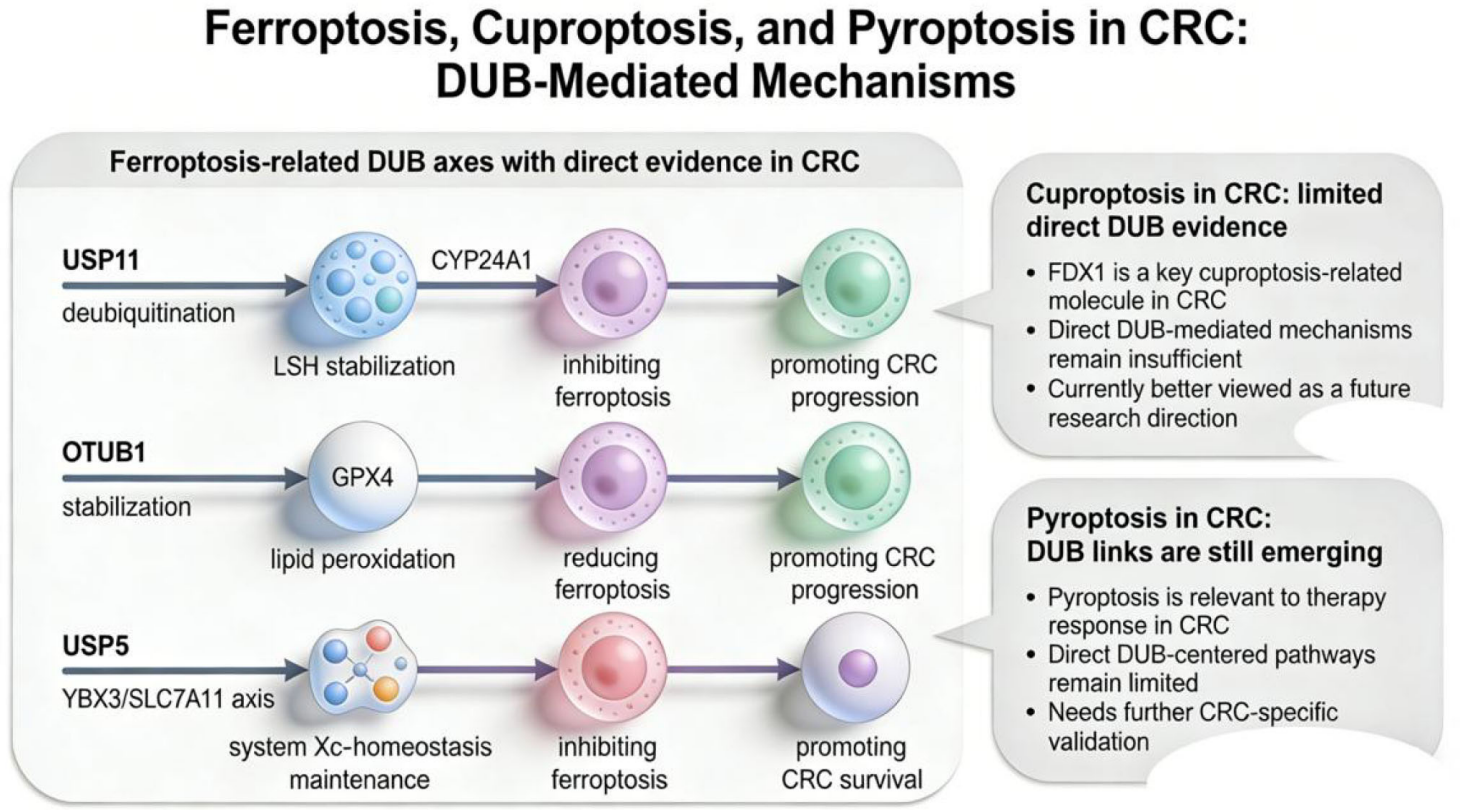
This figure primarily illustrates the relationship between DUBs and non-apoptotic cell death in CRC. The solid lines in the figure represent ferroptosis-related DUB pathways currently supported by relatively direct evidence in CRC, including USP11–LSH/CYP24A1, OTUB1–GPX4, and USP5–YBX3/SLC7A11. All three pathways are associated with reduced ferroptosis sensitivity and are linked to tumor progression or treatment resistance (33–35). In contrast, direct evidence for DUB regulation of copper-mediated cell death and pyroptosis in CRC remains limited; therefore, these are represented in the figure only by light gray callouts, presented as prospective research directions rather than being interpreted at the same level of evidence as the ferroptosis pathways (36) (Created by Figdraw).

Ferroptosis is a form of cell death that depends on ferrous ions (Fe²^+^) and is accompanied by lipid peroxidation. CRC cells can acquire resistance by regulating the expression and stability of ferroptosis-related molecules, thereby promoting tumor (33–35). It should be emphasized that the DUB-ferroptosis axis currently supported by the most direct evidence in CRC is not the USP7/USP14-GPX4/SLC7A11 pathway, but rather consists primarily of three pathways: First, USP11 inhibits ferroptosis by stabilizing LSH via the CYP24A1 axis, thereby promoting CRC progression; second, recent studies suggest that OTUB1 can inhibit ferroptosis by stabilizing GPX4; third, a 2025 study further suggests that USP5 regulates CRC sensitivity to ferroptosis via the YBX3/SLC7A11 axis (33–35). In contrast, USP7, USP14, and some NRF2-related pathways are primarily suggested as mechanisms at the pan-cancer level; when discussed in the context of CRC, they are more appropriately labeled as “speculative associations” rather than “confirmed in CRC” (4). Therefore, this paper classifies DUB-mediated regulation of ferroptosis into two categories: one consists of pathways directly validated in CRC models, such as USP11-LSH/CYP24A1, OTUB1-GPX4, and USP5-YBX3/SLC7A11; the other comprises pan-cancer inferred mechanisms that are biologically plausible but still require validation through direct CRC perturbation experiments (33–35) (**Figure 4**).

It is also important to note that DUB regulation of ferroptosis itself is subject to upstream chemical modifications (37). Existing colorectal cancer studies suggest that endogenous H_2_S can regulate the stability of xCT/SLC7A11 by inducing thiol-sulfide modification at the C91 site of OTUB1, indicating that “the modification status of DUB activity itself” also influences the ferroptosis threshold. In other words, future research on ferroptosis in CRC should not merely focus on “which DUB stabilizes which substrate,” but should further analyze under what redox, metabolic, and therapeutic stress conditions a given DUB is activated or inactivated (37). From a translational perspective, the next priority should not be to continue expanding the list of iron death-associated DUBs in parallel, but rather to clarify whether these DUBs truly determine the therapeutic susceptibility of CRC to oxaliplatin, radiotherapy, or iron death-inducing strategies.

### 5.2 Regulatory roles of DUBs in novel cell death modes (copper death and pyroptosis)

Copper death is a copper-dependent regulated form of cell death. Its core mechanism involves the binding of excess intracellular copper to acetylated proteins in the tricarboxylic acid cycle, leading to acetylated protein aggregation, loss of iron-sulfur cluster proteins, and protein toxic stress, ultimately resulting in cell death (38). The FDX1 protein is considered one of the key regulators in this process and is closely associated with copper ion reduction and acylation-related metabolic processes (**Figure 4**). In CRC, research on DUBs directly regulating copper-mediated cell death remains limited; existing studies in CRC have primarily focused on changes in FDX1 expression itself and its relationship with tumor progression (38, 39). For example, studies have shown that FDX1 expression is downregulated in CRC, and its upregulation can inhibit cell proliferation, migration, and the epithelial-mesenchymal transition (EMT) process, suggesting that copper-mediated cell death-related molecules may participate in regulating the biological behavior of CRC; however, this does not directly indicate that a specific DUB has been confirmed as a core regulator of copper-mediated cell death in CRC. Although studies in other cancer types suggest that deubiquitination processes may influence sensitivity to ferroptosis, this aspect remains in the exploratory stage in CRC. Therefore, this paper tends to view DUB regulation of ferroptosis as a direction worthy of attention and further research (38, 39).

Compared to ferroptosis, evidence for the direct regulation of pyroptosis in CRC by DUBs remains limited. Existing studies suggest that deubiquitination processes may indirectly contribute to the remodeling of the CRC immune microenvironment by influencing inflammasome assembly, NLRP3-related signaling, or GSDME-mediated therapy-induced pyroptosis. However, a significant portion of these mechanisms still primarily stems from immunological studies or other tumor models and cannot yet be regarded as established DUB pathways in CRC. Based on current evidence, findings related to copper death and pyroptosis in CRC are better suited to be proposed as hypotheses for generating new research and as directions for prospective studies, rather than being understood at the same level of maturity as the ferroptosis pathway (36, 38, 40, 41).

Summary of this section: In the field of non-apoptotic cell death, the DUB axis in CRC, for which relatively direct evidence is available currently, focuses primarily on ferroptosis; in contrast, mechanisms related to copper-mediated cell death and pyroptosis remain largely at the inferential or prospective level (33–35). This suggests that the maturity of evidence for different cell death pathways in CRC is inconsistent. At this stage, the approach with greater translational value is not to pursue all novel cell death mechanisms simultaneously, but rather to prioritize the ferroptosis axis—for which direct evidence already exists—and further evaluate its association with oxaliplatin, radiotherapy, and treatment-induced stress vulnerability. A priority for future research should be to clarify which DUBs truly determine CRC’s sensitivity to ferroptosis-related interventions and which serve primarily as co-regulatory factors, thereby providing a clearer mechanistic basis for combination therapy strategies.

## 6 Regulatory roles of DUBs in the CRC immune microenvironment

### 6.1 Intrinsic immune evasion mechanisms of CRC tumor cells

Regarding immune checkpoint regulation, the abnormally high expression of programmed death-ligand 1 (PD-L1) on the surface of CRC cells is one of the key pathways enabling intrinsic immune evasion by tumor cells (36, 42, 43). Previous studies have shown that various DUBs can directly participate in maintaining PD-L1 protein stability, a finding supported by CRC models and some clinical samples (7, 36, 42, 43). For example, USP22 directly binds to PD-L1 in CRC and reduces its ubiquitination levels; recent studies also suggest that the small molecule demethylase zeylasteral can lower PD-L1 levels by promoting the degradation of USP22 (36, 42). Similarly, USP7 can also stabilize PD-L1 in CRC and has demonstrated potential for combination with anti-programmed death receptor-1 (PD-1) therapy in organoids and patient-derived xenograft (PDX) models (43). However, the efficacy of such approaches remains to be determined. Effective antitumor immune responses depend on the recognition of tumor neoantigens by cytotoxic CD8^+^ T cells, a process that requires major histocompatibility complex class I (MHC-I) molecules to present antigenic peptides. It should be noted that these mechanisms are better described as “PD-L1 stabilization mechanisms reported in CRC models” rather than broadly characterized as “CRC-specific regulation,” as there is currently insufficient cross-cancer comparative evidence to prove their exclusivity to CRC (36, 42, 43). In terms of the maturity of the evidence, the PD-L1 stabilization axis remains the DUB pathway with the most direct evidence and the closest proximity to translational discussions regarding intrinsic immune evasion in CRC tumor cells.

In addition to immune checkpoints, intrinsic antigen processing and presentation in tumor cells also determine their immunological visibility and transport to the tumor cell surface. Existing studies suggest that, in addition to PD-L1 stabilization, DUBs may also participate in intrinsic immune evasion by influencing interferon (IFN) signaling and processes related to antigen processing and presentation; however, based on current direct evidence in CRC, this aspect remains generally weaker than the PD-L1 stabilization axis (7). Furthermore, the STING/IRF3, NF-κB, and antigen-presentation-related pathways also contribute to understanding how DUBs reshape the immunological visibility of CRC (7, 44).

A 2024 study demonstrated that USP4 is upregulated in microsatellite-stable (MSS) CRC and, by removing K63-linked ubiquitin chains from TRAF6 and IRF3, inhibits IRF3 nuclear translocation, attenuates the intrinsic IFN response in tumor cells, downregulates antigen presentation capacity, and reduces pattern recognition receptor-associated cell death (7). This finding suggests that the impact of DUBs on CRC immune evasion is not limited to immune checkpoints but also involves alterations in innate immune signaling and antigen presentation, providing molecular clues to explain the limited response of MSS CRC to immune checkpoint monotherapy. In contrast, NF-κB-associated DUB regulation in CRC currently manifests primarily as interactions surrounding the TRAF6 axis; therefore, this study tends to regard it as a concomitant pathway of immune remodeling rather than a fully established universal mechanism in CRC (7).

Overall, research on DUBs associated with intrinsic immune evasion in tumor cells has formed two main lines of inquiry in CRC, each with a different level of evidence: The first line involves the PD-L1 stabilization axis represented by USP22 and USP7, which offers more direct evidence and is easier to integrate with immune checkpoint therapy to form a translational rationale; The second tier is the IFN/antigen visibility regulation axis, represented by USP4, whose significance lies in suggesting that DUBs may further influence the “immunological visibility threshold” of CRC. Moving forward, the priority should not be to continue expanding the list of PD-L1-stabilizing DUBs, but rather to identify which DUBs can effectively address both key limiting factors: immune checkpoint dependence and insufficient antigen presentation.

### 6.2 DUB-mediated exogenous functional remodeling of immune cells in the tumor microenvironment

**Figure 5:**
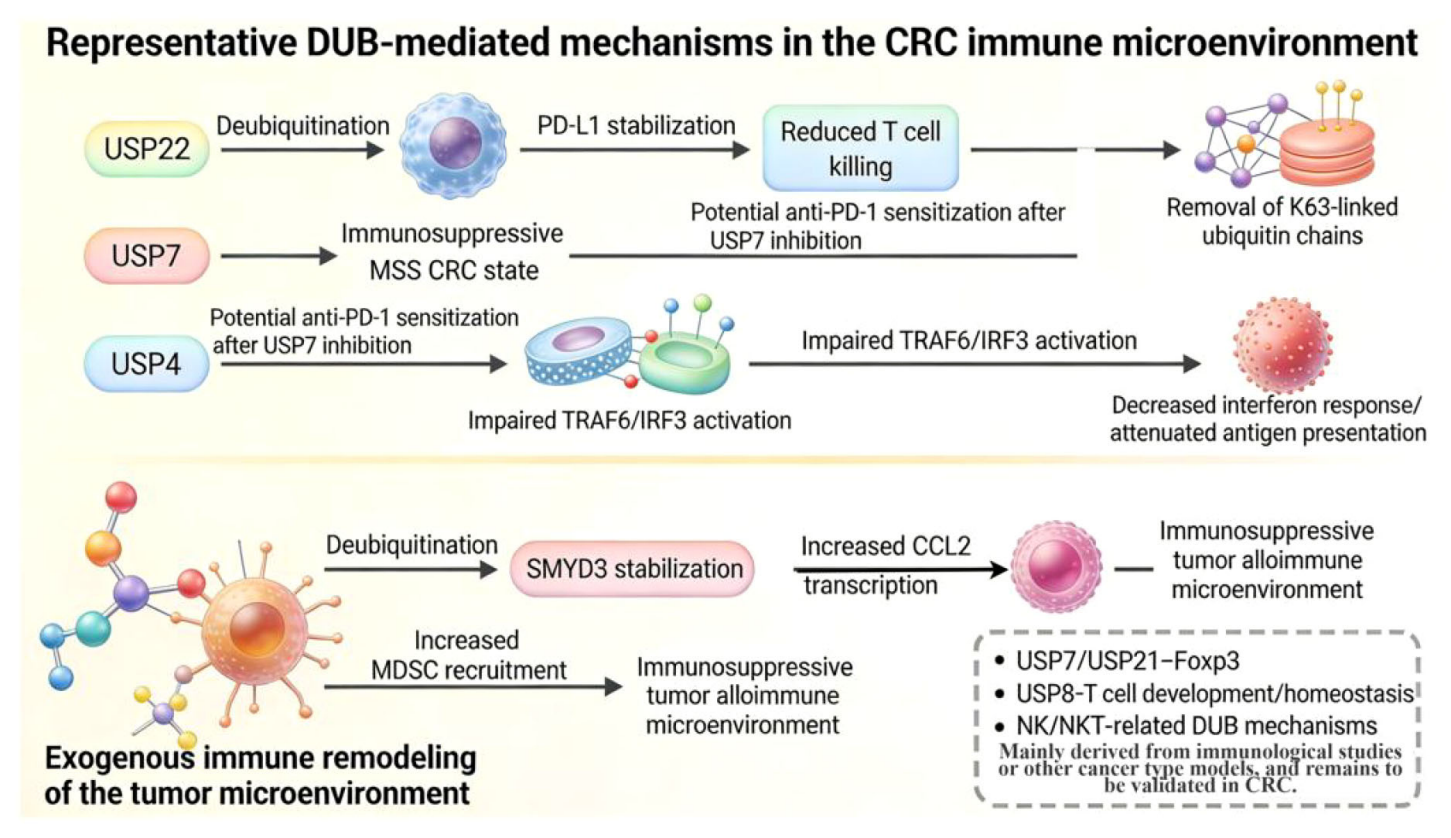
Representative regulatory mechanisms of DUBs in the CRC immune microenvironment. Solid lines indicate associations currently supported by relatively direct evidence in CRC, including the USP22–PD-L1 stabilization axis, USP7 and its association with the immunosuppressive state of MSS CRC and potential for combination with anti-PD-1, the USP4–TRAF6/IRF3–interferon response/antigen presentation axis, and the USP15–SMYD3/CCL2–MDSC axis. Dotted gray boxes denote inferential mechanisms primarily derived from immunological studies or models of other cancer types, such as Treg stabilization, T cell homeostasis, and NK/NKT-related DUB functions; these mechanisms require further systematic validation in CRC. This figure aims to highlight that the maturity of evidence varies across different levels (7, 32, 36, 42, 43, 45–49). (Created by Figdraw).

In addition to intrinsic immune evasion by tumor cells, DUBs may also indirectly shape the immunosuppressive landscape of CRC by influencing the state of immune cells in the tumor microenvironment. However, the evidence supporting this mechanism is generally less robust than that for intrinsic tumor cell mechanisms. Current studies suggest that certain DUBs are involved in the maintenance of regulatory T cell (Treg) homeostasis, T cell receptor signaling, and the function of innate immune cells (45–48). For example, USP7 and USP21 are associated with Foxp3 protein stability, while USP8 is involved in T cell development and homeostasis maintenance; however, these findings primarily stem from immunological studies or non-CRC models, and systematic validation of the same caliber is still lacking in CRC (46–48)(**Figure 5**). Therefore, this body of evidence is better regarded as “external evidence providing insights into the CRC immune microenvironment” rather than a direct mechanism fully established in CRC.

In contrast, direct evidence regarding myeloid immune suppression in CRC is more robust. A 2025 study demonstrated that USP15 is highly expressed in CRC and can stabilize SMYD3 through deubiquitination, thereby enhancing H3K4me3-dependent CCL2 transcription and promoting the recruitment of myeloid-derived suppressor cells (MDSCs); in a cohort of patients receiving immunotherapy, high USP15 expression was also associated with poorer anti-PD-1 responses, suggesting that certain DUBs not only contribute to the formation of the immunosuppressive microenvironment in CRC but may also hold predictive value for treatment response, warranting particular attention in microsatellite-stable/mismatch repair-proficient (MSS/pMMR) CRC (45).

In terms of the hierarchy of evidence, research on DUBs associated with exogenous immune remodeling in CRC has not yet established a main axis as robust as the intrinsic mechanisms of tumor cells. More precisely, the clearest direct evidence in this field currently centers on the USP15–SMYD3–CCL2–MDSC myeloid suppression axis, while DUB mechanisms related to Treg homeostasis, T-cell development, and innate immune cells remain largely at the stage of “biologically plausible but lacking direct evidence of disruption in CRC” (45–48). This does not imply that these directions are unimportant; rather, it suggests that, at this stage, review writing should place greater emphasis on the boundaries of evidence, rather than treating all exogenous immune regulatory mechanisms as being at the same level of maturity.

Therefore, based on the existing literature, DUBs may indeed participate in the remodeling of the CRC microenvironment through intrinsic mechanisms of immune cells; however, the most clinically relevant clues regarding exogenous immune remodeling in CRC at this stage remain primarily concentrated on the USP15–SMYD3–CCL2–MDSC axis (45) (**Figure 5**). In the future, priority should be given to establishing higher-level direct evidence for CRC, specifically regarding myeloid suppression, low response to immunotherapy, and the MSS/pMMR context, rather than prematurely extrapolating pan-cancer immunological conclusions to CRC as a whole.

### 6.3 DUBs as a hub regulating intrinsic tumor survival and extrinsic immune evasion in CRC

Overall, DUBs do not act in isolation in CRC. On the one hand, they enhance the survival advantage of tumor cells by regulating DNA damage repair, metabolic adaptation, and cell death thresholds; on the other hand, they contribute to immune evasion by influencing processes such as PD-L1 stability, interferon responses, and myeloid suppression. Therefore, DUBs are better understood as regulatory nodes linking intrinsic stress adaptation in tumor cells to external immune suppression, rather than as molecules in a single pathway. It is important to note that the evidence supporting this “bridge function” is not entirely balanced across different levels: while intrinsic mechanisms in tumor cells have been extensively studied in CRC, a significant portion of the evidence regarding intrinsic mechanisms in immune cells still originates from immunological studies or other tumor models (7, 32, 36, 42, 43, 45).

Existing CRC studies provide direct support for this integrated understanding. For example, USP22 stabilizes PD-L1 through deubiquitination, thereby enhancing the immune evasion capacity of tumor cells; USP4 suppresses IRF3 activation, the intrinsic interferon response in tumor cells, and antigen presentation-related processes by removing K63-linked ubiquitin chains from TRAF6 and IRF3; while USP14 promotes tryptophan metabolism and immune suppression by stabilizing IDO1. These findings suggest that DUBs not only enhance the survival and adaptability of CRC cells but may also simultaneously weaken the host’s antitumor immune response (7, 32, 36, 42, 43, 45) (**Figure 5**). In this sense, the role of DUBs in CRC is not simply distributed across parallel modules such as “metabolism,” “DNA repair,” and “immune evasion,” but rather links these processes together by influencing a few key nodes.

Taking USP7 as an example, its integrative role warrants careful interpretation. CRC studies suggest that USP7 is associated with an immunosuppressive state in MSS CRC and may serve as a potential immunotherapy target; conversely, immunological research has demonstrated that USP7 participates in Foxp3 stabilization within Tregs (43, 46). It can thus be inferred that USP7 may collectively promote immunosuppression across different cell populations; however, this integrative model still lacks direct validation of equivalent caliber in CRC (43, 46). Therefore, at this stage, a more reasonable approach is not to directly describe USP7 as a “proven central hub with dual roles in both tumor cells and immune cells,” but rather to regard it as a representative DUB with the greatest potential for integration, though further validation in higher-level CRC models is still required.

In summary, DUBs in CRC appear to function more as key regulatory nodes connecting the intrinsic survival programs of tumor cells with the external immunosuppressive environment. This understanding helps explain why interventions targeting a single DUB often yield limited results and suggests that a more rational future strategy may not be to treat DUBs as monotherapy targets, but rather to consider them within a tiered combination framework involving chemotherapy, targeted therapy, or immunotherapy (7, 32, 36, 42, 43, 45). Furthermore, for CRC, the truly critical next step is not to continue expanding the list of immune-related DUBs, but to identify which DUBs can effectively shift the MSS/pMMR background from a state of low antigenic visibility and low responsiveness back to a more responsive immune ecosystem. This may hold greater translational significance than simply expanding the single-mechanism axis itself.

Summary of this section: The impact of DUBs on the CRC immune microenvironment extends beyond a single immune checkpoint, involving multiple levels such as intrinsic immune evasion by tumor cells, reduced antigen accessibility, and exogenous myeloid suppression. Based on current direct evidence in CRC, the USP22/USP7-associated PD-L1 stabilization axis, the USP4-associated IFN/antigen presentation axis, and the USP15–SMYD3–CCL2–MDSC axis constitute relatively more mature pathways; in contrast, Treg homeostasis and other intrinsic DUB mechanisms in immune cells still rely primarily on support from pan-cancer or immunological studies. Thus, DUBs in CRC appear to function as bridge-like nodes connecting the intrinsic survival advantages of tumor cells with the external immunosuppressive environment. Moving forward, the priority should not be to continue expanding the list of immune-related DUBs, but rather to identify which DUBs can truly enhance the immunoresponsiveness of MSS/pMMR CRC and, based on this, establish clearer stratified combination therapy strategies.

## 7 Clinical translation: from traditional inhibitors to PROTAC technology innovation

### 7.1 Current status and challenges of small-molecule inhibitors

In recent years, the development of small-molecule inhibitors targeting deubiquitinating enzymes (DUBs) has continued to advance, but the number of drug candidates truly nearing clinical translation remains limited. Currently, the most prominent targets include USP7, USP1, USP14, and USP22, among others (4, 8). It should be noted that the significance of DUB inhibitors in CRC at this stage is no longer merely about “whether candidate drugs exist,” but rather whether these drugs can demonstrate reproducible efficacy, explainable mechanisms of action, and actionable patient stratification strategies within a well-defined molecular context. In other words, the key issue currently facing the CRC field is not a shortage of DUB inhibitors but rather the lack of a sufficiently clear framework regarding target-dependent contexts, combination therapy logic, and translational pathways.

From a target perspective, USP7 is one of the most extensively studied yet also the most context-dependent candidate DUBs. Existing CRC-related studies have shown that the USP7 inhibitors P5091 and FT671 exhibit certain preclinical activity; however, the effects of these drugs cannot be reliably extrapolated to all CRC models. This is because USP7 is not a “linear target” with a single function, but rather sits at the intersection of multiple substrate networks: the key downstream pathways regulated by USP7 are not entirely the same across different TP53 statuses, APC/Wnt backgrounds, and immune microenvironments. More importantly, existing studies have suggested that USP7 inhibition is more likely to yield detectable efficacy in the context of APC truncation/high Wnt activation or TP53 mutations: the former is associated with β-catenin deubiquitination, while the latter is related to mutant p53 and the maintenance of a cancer stem-like state. Therefore, USP7 inhibition in CRC is better understood as a “context-dependent combination therapy target” rather than a monotherapy strategy universally applicable to all CRC cases (5–8).

Compared to USP7, the therapeutic role of USP1 is relatively clearer. The USP1 inhibitor ML323 primarily acts on the FANCD2/PCNA-related DNA damage repair axis in CRC; its biological significance lies more in enhancing sensitivity to DNA damage therapy rather than directly inhibiting tumor growth as an independent monotherapy. This actually points to a more realistic application logic for DUB inhibitors in CRC: the value of certain targets lies not in “direct killing,” but in amplifying the replicative stress or DNA damage caused by existing treatments. RO7623066 (KSQ-4279), a selective USP1 inhibitor that has entered Phase I clinical trials, further demonstrates that the USP1 pathway has progressed from a tool compound to the clinical exploration stage (10); However, it should also be noted that its development is not exclusive to CRC but rather aligns more closely with DNA damage response-dependent therapeutic strategies in a pan-cancer context. Therefore, with regard to CRC, USP1 inhibition is currently better understood as a “translational target with a clear logic for combination therapy” rather than a mature strategy with sufficient evidence for monotherapy (9).

In contrast, although USP14 and USP22 are associated with tryptophan metabolism/immune suppression and PD-L1 stabilization and lipid metabolism, respectively, in CRC, the development of their inhibitors lags significantly behind that of USP1 and USP7. On the one hand, the biological functions of these targets are highly context-dependent; on the other hand, their translational value is more likely to manifest in specific therapeutic combinations rather than through simple monotherapy. USP22, in particular, is involved in both immune evasion and epigenetic and lipid metabolism in CRC. While this “multi-layered connectivity” enhances its theoretical appeal, it also increases the complexity of interpreting efficacy and assessing toxicity. For these targets, the current greatest need is not for more *in vitro* inhibition experiments, but for systematic validation centered on specific models, particular substrates, and pharmacodynamic biomarkers (5–8).

From a drug-tiering perspective, current DUB/UPS-related candidate molecules can be broadly categorized into three groups. The first group consists of preclinical tool compounds, such as the USP7 inhibitors P5091 and FT671, and the USP14 inhibitor IU1 series. These molecules are better suited for validating target druggability, defining therapeutic windows, and identifying potential companion biomarkers. The second category consists of selective candidate molecules that have entered the clinical exploration phase, represented by the USP1 inhibitor RO7623066 (KSQ-4279). The significance of this lies in demonstrating that certain DUB targets can progress from mechanism validation to clinical development. The third category consists of molecules that are not DUB inhibitors but are of comparative value in UPS pharmacology, such as the ubiquitin-activating enzyme UBA1 (E1) inhibitor TAK-243: Since TAK-243’s true target is not USP1 or any other DUB, it can be discussed as a comparative agent for UPS intervention but should no longer be classified as a DUB/USP1 inhibitor (4, 8, 10, 50, 51).

Beyond target selection, another major practical challenge for DUB inhibitors lies in balancing selectivity, toxicity, and pharmacokinetics/pharmacodynamics (PK/PD). The previous clinical discontinuation of VLX1570 has already indicated that even with strong *in vitro* activity, DUB inhibitors struggle to reach a truly clinically viable stage if they lack sufficient selectivity, appropriate formulation characteristics, and an acceptable safety margin (51). This is particularly critical for CRC, as many DUB candidates are involved in DNA repair, immune homeostasis, and metabolic adaptation; their inhibitory effects may not be confined to tumor cells but could also impact stress responses in normal tissues and immune balance. Therefore, what truly deserves priority in the future CRC field is not the continued expansion of the list of DUB inhibitors, but the establishment of a more concrete translational framework centered on “target-dependent context—combination therapy logic—biomarkers of efficacy—toxicity window”.

Based on the existing literature, this paper tends to argue that the realistic near-term positioning of DUB small-molecule inhibitors in CRC is not as broad-spectrum new drugs for all patients, but rather as combination therapy enhancers or vulnerability amplifiers under specific molecular backgrounds. In terms of translational priorities, USP1 aligns more closely with a direction characterized by a clear mechanism and well-defined combination logic; USP7 represents a direction with strong biological appeal but higher requirements for context-dependence and patient stratification; as for other targets such as USP14 and USP22, they are currently better suited as extensions for mechanism and combination strategy exploration rather than as mature monotherapy pathways with established clinical feasibility (**Table 1**).

### 7.2 Complementary strategies of PROTACs and DUBTACs

Proteolytic Targeting Assemblies (PROTACs) and Deubiquitinating Enzyme Targeting Assemblies (DUBTACs) represent a more platform-oriented technological evolution within DUB-targeting strategies. Unlike traditional small-molecule inhibitors, which primarily rely on occupying catalytic sites, PROTACs induce target protein degradation by simultaneously binding to both the target protein and an E3 ubiquitin ligase; DUBTACs, on the other hand, aim to recruit specific DUBs to the vicinity of the target protein, thereby preventing its degradation and restoring its function by removing ubiquitin modifications (52). Although these two strategies appear to take opposite directions, they are pharmacologically complementary: the former is suitable for targeting oncogenic proteins, particularly those with both enzymatic and non-enzymatic functions; the latter is better suited for stabilizing proteins that play beneficial roles in tumor suppression, stress adaptation, or treatment sensitivity.

In CRC research, the appeal of PROTACs lies primarily in their theoretical ability to address DUB biology that traditional inhibitors struggle to address fully. Taking USP7 as an example, USP7 not only possesses enzymatic activity but also frequently assumes scaffold-like functions through substrate networks and protein interactions; therefore, simply inhibiting its catalytic activity may not fully eliminate its oncogenic effects. The emergence of USP7 PROTAC molecules such as U7D-1 has, at least conceptually, demonstrated that USP7 is “degradable” (50). However, it must be emphasized that existing evidence of this kind primarily stems from non-CRC tumor cells or early-stage, pan-cancer pharmacological studies, particularly focusing on degradability and antiproliferative activity in p53-related or p53-mutated cancer cells; this cannot yet be equated with higher-level, direct evidence established in CRC. Therefore, while the USP7 degrader demonstrates that DUBs can be incorporated into degradation-based therapeutic strategies, whether it outperforms traditional inhibitors in CRC remains to be further validated in models more closely aligned with the disease context.

The DUBTAC approach, however, exhibits more of a “reverse regulation” characteristic. Unlike PROTACs, which degrade pathogenic proteins, DUBTACs aim to stabilize target proteins by recruiting DUBs, thereby restoring protein function. This strategy is theoretically well-suited for addressing tumor suppressor proteins or treatment-sensitivity-related proteins that are degraded due to excessive ubiquitination, suggesting significant potential. More importantly, the DUBTAC platform is not confined to the purely theoretical realm: early prototype studies have demonstrated that an OTUB1-recruiting DUBTAC can stabilize ΔF508-CFTR, while subsequent TF-DUBTAC research has further shown that this strategy can stabilize transcription factors such as p53, FOXO3A, and IRF3 under OTUB1-dependent conditions. These results indicate that the significance of DUBTACs lies not merely in “preventing the degradation of a specific protein,” but rather in providing a chemical stabilization pathway for proteins that are functionally impaired but not completely inactivated (52–54). For CRC, the most promising future application of this approach may not be the direct stabilization of a traditional oncogene, but rather the restoration of key proteins that determine drug response, immune visibility, or sensitivity to cell death.

However, it must be acknowledged that DUBTACs are currently still clearly in the early proof-of-concept stage. The number of DUBs that can be stably recruited is limited, suitable ligand resources are insufficient, the mechanisms governing ternary complex formation are not yet well-established, and systems for cellular permeability, *in vivo* delivery, structure-activity relationships, and pharmacodynamic biomarkers are far from mature (52–54). Existing reviews also point out that, although OTUB1 and USP7 have been utilized in DUBTAC design, the overall DUBTAC toolkit remains very limited; meanwhile, the 2025 work on USP1-recruiting DUBTACs primarily reflects the platform significance of “further expanding the range of recruitable DUBs,” rather than indicating that CRC already possesses mature, readily available protein stabilization strategies. Therefore, for CRC, DUBTACs are currently better suited as a promising platform reserve rather than a mature pathway with established clinical feasibility (52–54).

Furthermore, the challenges faced by both PROTACs and DUBTACs extend beyond merely “whether bifunctional molecules can be designed” to whether stable, predictable, and pharmacologically viable ternary complexes can be formed. For PROTACs, common issues include high molecular weight, limited oral exposure, insufficient tumor penetration, suboptimal linker configuration, the “hook effect,” and mismatches between tissue distribution and pharmacodynamic biomarkers (52–54). For DUBTACs, the challenges are even more fundamental: the currently available DUB-recruiting ligands are limited, and there are significant differences in cellular localization, substrate preferences, and functional backgrounds among different DUBs. Furthermore, if “inhibitory DUB ligands” are used, potential off-target toxicity may be incorporated into the stabilization strategy. In other words, the true bottlenecks for these two technologies currently lie not in the novelty of the concept, but rather in whether pharmacological controllability, tissue-level delivery capabilities, and *in vivo* efficacy evaluation systems are sufficiently mature (52–54).

For CRC, these technical challenges have a specific context. CRC is a highly heterogeneous solid tumor characterized by significant differences in location, molecular subtypes, and immune microenvironments. Theoretically, this heterogeneity offers opportunities for tiered application of PROTACs and DUBTACs; however, the local intestinal environment, tissue permeability, spatial heterogeneity within tumors, and differences in the expression profiles of various E3 ligases or DUBs also make these platform technologies more susceptible to delivery and pharmacokinetic constraints in CRC. Existing studies on nano-PROTACs and self-assembling PROTACs suggest that improving exposure and tissue distribution in solid tumors through delivery systems may be a crucial step toward truly integrating these technologies into translational CRC research. Therefore, rather than being an ideal testing ground for PROTACs/DUBTACs, CRC represents a complex application scenario that presents both opportunities and significant challenges.

Overall, PROTACs are used to degrade oncogenic DUBs, while DUBTACs are used to stabilize potentially beneficial proteins; these two strategies are pharmacologically complementary. However, in CRC, they currently remain primarily in the early stages of proof-of-concept and platform exploration (50, 52–54). Based on the current evidence, this paper tends to view the most realistic significance of these two technologies in the short term not as the rapid development of new CRC drugs ready for clinical use, but rather as a means to help researchers overcome the limitations of traditional inhibitors in terms of functional coverage and selectivity, while providing a technological foundation and platform support for more precise, stratified combination therapies in the future. To avoid simply listing different targets and platform approaches side by side, this paper further conducts a comparative analysis of representative DUB/UPS-related strategies across three dimensions: level of evidence, major limitations, and more appropriate positioning for CRC applications (**Table 2**).

**Table 2:** Current status of translation, key limitations, and more appropriate clinical applications of CRC-related DUB-targeting strategies.

Target/Platform	Representative molecules or strategies	Mechanism of action	Levels of evidence	CRC: Relevant applications/target audience	Current development stage	Main limitations	A more reasonable approach to CRC localization at this stage
USP1 (4, 6, 52)	ML323	Small-molecule inhibitors	CRC: Direct preclinical evidence	DDR-dependent, platinum-based/sensitization to replication-stress-related therapies.	Preclinical	Insufficient evidence for monotherapy; potential toxicity due to DNA repair dependence in normal hyperproliferative tissues.	It is better suited as a combination sensitizer for DNA damage therapy to enhance the therapeutic sensitivity of DDR-dependent CRC, rather than as a monotherapy target with well-established evidence.
USP1 (4, 10)	RO7623066(KSQ-4279)	Selective small-molecule inhibitors	Pan-cancer clinical evidence; not limited to CRC	DDR-dependent subgroups in advanced solid tumors; CRC may serve as one of the candidate indications.	Phase I clinical trial	No specific clinical data on CRC is currently available; there is insufficient information on accompanying biomarkers and the definition of the target patient population.	This falls under the USP1 pathway, which has already entered pan-cancer clinical trials and holds potential translational value for CRC; however, at this stage, it still relies on combination therapy regimens to identify the appropriate patient population.
USP7 (4, 5, 7, 8, 19, 55, 56)	P5091	Small-molecule inhibitors	CRC Preclinical Evidence	TP53 abnormalities, APC/Wnt dysregulation, or an immunosuppressed state.	Preclinical	Broad substrate range; high background dependence; complex interpretation of selectivity and potency.	More suitable as a context-dependent co-target, with priority given to its use in CRC under conditions of inactivation or immunosuppression.
USP7 (4, 8, 57)	FT671	Highly selective small-molecule inhibitor	Preclinical - Mechanism Validation	It is more suitable for validating the molecular window dependent on USP7 rather than directly extrapolating the entire CRC.	Preclinical	High-level direct evidence remains limited; the specific subgroups of interest have yet to be identified.	At this stage, it is more appropriate to position this compound as a highly selective pharmacological tool for defining USP7-dependent subgroups, rather than directly extrapolating it as a mature treatment regimen for CRC.
Comparison of UPS Drugs (11, 52, 58)	TAK-243	UBA1/E1 inhibitor	Pan-cancer Clinical - Preclinical or Exploratory Clinical	Drugs that can be used as comparators for UPS-wide interventions.	Non-DUB comparator drugs	This is not a DUB inhibitor and should not be mistakenly classified under the USP1 pathway.	This should be used solely as a comparative reference for overall UPS interference; it should no longer be classified under the DUB or USP1 inhibitor pathways, nor should it be used as evidence for DUB targeting in CRC.
USP7 Degradation Strategy (50, 53, 59, 60)	U7D-1 and others, such as USP7 PROTAC	PROTAC Degradant	Early preclinical stage, primarily focused on a pan-cancer proof-of-concept for non-CRC	It is difficult to characterize the full range of DUB functions based solely on enzyme activity inhibition.	Early-stage platform validation	Insufficient direct evidence for CRC; delivery and pharmacokinetics remain limited.	There are currently no established treatment options for CRC.
DUBTAC Platform (53, 54)	OTUB1-recruiting TF-DUBTAC; USP1-recruiting DUBTAC	Targeted Stabilization Platform	Platform-level early evidence, primarily non-CRC	Proteins involved in stabilizing tumor suppressors or restoring sensitivity to treatment.	Proof of Concept - Early-Stage Platform	The pool of available DUBs is limited; structure-activity relationships, delivery systems, and *in vivo* pharmacodynamic systems are not yet fully developed.	At this stage, it is more suitable as an early-stage technological foundation and protein stabilization platform for expanding the space of druggable targets in CRC; a mature, clinically viable treatment pathway has not yet been established.
Evidence Supporting the CRC Delivery Platform (53, 60)	self-assembling PROTAC	Delivery/Platform Optimization Strategies	CRC Preclinical Evidence	Enhance PROTAC exposure, tissue distribution, or TME reprogramming capabilities in solid tumors.	Preclinical	This demonstrates delivery feasibility; it does not prove that a specific DUB target is ready for development.	This serves as further technical evidence supporting the improvement of PROTAC delivery in solid tumors, highlighting the necessity of platform optimization in CRC.

This table is not intended merely to list representative molecules; rather, it summarizes the practical translational status of various DUB/UPS-related strategies in CRC by integrating the current level of evidence, applicable contexts, key limitations, and comprehensive assessments.

### 7.3 Synergistic enhancement and synthetic lethality combination therapy strategies

Since monotherapy targeting a single DUB is susceptible to compensatory pathways, and given the significant heterogeneity of CRC itself, combination therapy and synthetic lethality strategies remain more realistic translational approaches (4, 8). In terms of enhancing the efficacy of chemotherapy, the most well-established application of DUB inhibitors remains combination therapy (4, 8). Inhibition of USP1 can disrupt the FANCD2- and PCNA-related DNA repair axis, thereby increasing CRC sensitivity to platinum-based drugs or PARP inhibitors at the mechanistic level; however, this effect is currently primarily supported by preclinical evidence (9). Regarding USP7, its potential value should not be simplistically summarized as “restoring p53 function,” but rather understood as: under different p53 genotypes and related substrate contexts, USP7 inhibition may attenuate tumor survival advantages through diverse pathways (5, 6, 8). Similarly, targeting DUBs such as USP22 and USP14 may also influence cancer stemness, immune suppression, and stress adaptation; however, the optimal indications and combination partners still require further clarification (12, 32, 42).

**Figure 6:**
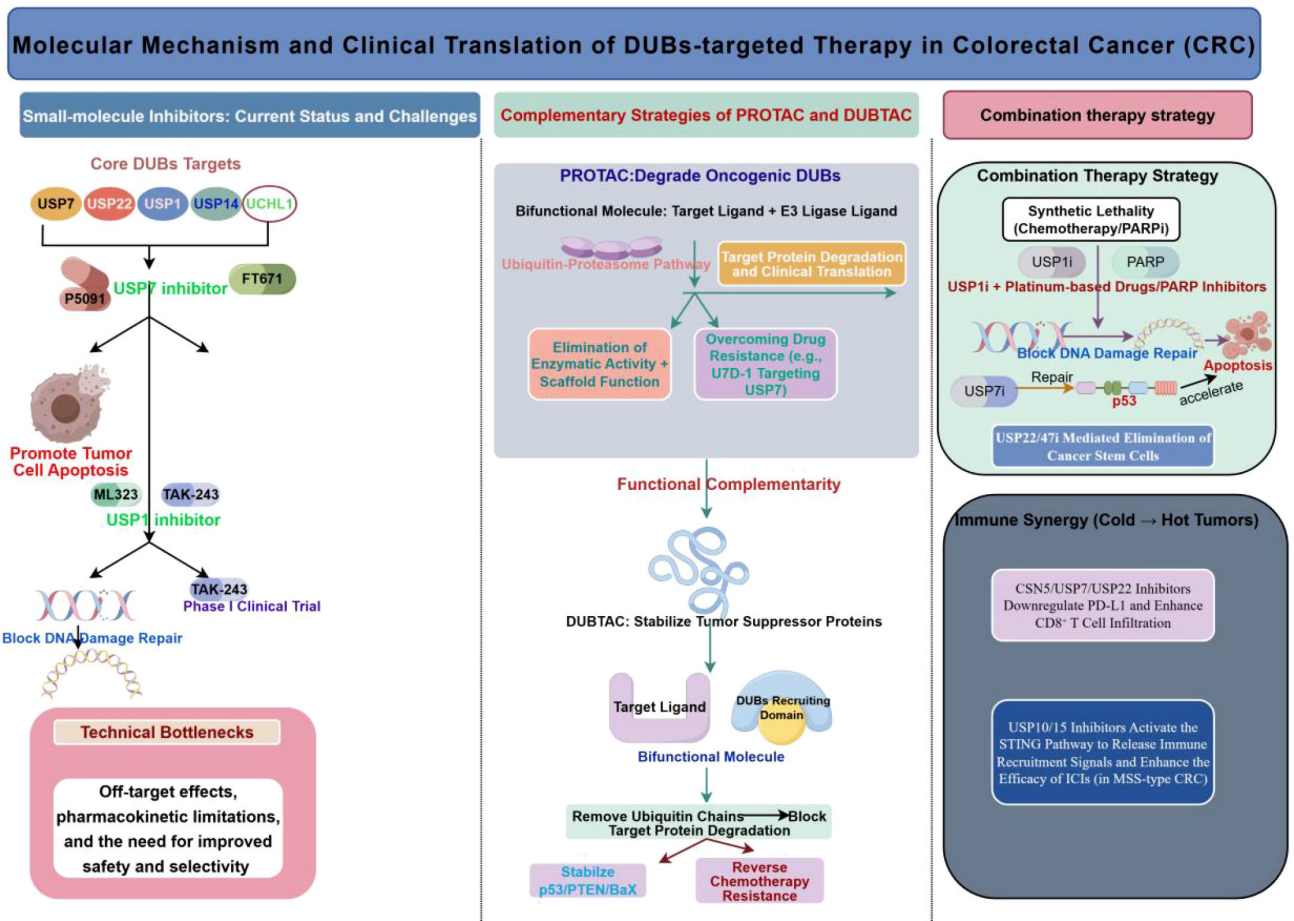
Major therapeutic approaches and combination strategies for DUB-targeted therapy in CRC. Solid lines indicate strategies supported by direct evidence in CRC, including USP1 inhibition to enhance sensitivity to DNA-damaging therapies and potential synergies between USP7-targeted strategies and anti-PD-1 therapy; Dotted lines indicate platform-based or pan-cancer inferences, including USP7 PROTACs, protein stabilization mediated by DUBTACs, and combination therapy concepts centered on USP15 and other immune-related DUBs (7, 9, 50, 52–54, 59, 60). TAK-243 is a UBA1/E1 inhibitor and is included solely as a comparator drug for UPS; it is not classified as a DUB inhibitor. (Created by Figdraw).

In terms of immunotherapy, DUB-targeting strategies hold some appeal for “cold” CRC subtypes such as MSS, but their true translational value is more likely to stem from “stratification + combination therapy” rather than widespread monotherapy (7, 46). For example, USP7 and USP22 are associated with PD-L1 stability; therefore, their inhibition or degradation is expected to complement the mechanism of PD-1/PD-L1 blockade. USP14, on the other hand, can link metabolic immunosuppression through IDO1 and tryptophan metabolism (7, 12, 42, 43). It should be noted that CSN5 is not a classical DUB but rather an UPS regulator adjacent to the DUB network; it should be distinguished from true DUB targets in discussions (**Figure 6**). Furthermore, the hypothesis that targets such as USP10 and USP15 activate innate immune alarm pathways and drive the transformation of “cold” tumors into “hot” tumors currently requires further direct evidence from CRC studies and is better suited as a direction for prospective research.

Regarding MSS/pMMR CRC, a key issue is not the addition of a new target, but whether tumors can be shifted from a state of “low antigen presentation, low interferon, and dominant myeloid suppression” back to a state more responsive to treatment. Based on current evidence, the synergy between DUB inhibition and immune checkpoint blockade is more likely to arise from four aspects: reducing PD-L1 stability (USP7/USP22 axis), restoring IRF3/IFN signaling and antigen accessibility (USP4 axis), alleviating CCL2-MDSC-mediated myeloid suppression (USP15 axis), and preventing DUBs from re-establishing immune tolerance following immunogenic damage induced by chemotherapy or radiotherapy (54) (**Figure 6**). In other words, the practical significance of DUB-targeting strategies for MSS CRC lies in reshaping the immune landscape rather than replacing existing immune checkpoint inhibitor regimens (46).

Summary of this section: The focus of DUB modification research has shifted from which inhibitors are available to which CRC subgroups, combination partners, and pharmacodynamic biomarkers are most suitable for prioritized development (4, 51–54). Therefore, the development of selectivity, pharmacokinetics/pharmacodynamics (PK/PD), companion diagnostics, and combination therapy designs should proceed in parallel with target identification.

### 7.4 The potential of DUBs as biomarkers for the diagnosis, prognosis, and prediction of treatment response in CRC

In addition to serving as therapeutic targets, DUBs also hold potential as biomarkers for CRC. Current evidence suggests that UCHL1 is associated with lymph node metastasis and may serve as a candidate biomarker for risk stratification; high expression of XAB2 is associated with poor prognosis and oxaliplatin resistance, making it a potential indicator for monitoring treatment response and resistance; high expression of USP15 is associated with poor response to anti-PD-1 therapy, suggesting its potential for inclusion in the companion diagnostic framework for immunotherapy; whereas USP22, USP7, and USP4 are better suited for combination with PD-L1, interferon response, and immune infiltration characteristics to form composite predictive models (7, 20, 42, 44, 46, 61).

However, it must be emphasized that evidence for most DUB biomarkers remains limited to retrospective tissue-level studies, lacking validation through prospective, multicenter trials and confirmation of functional and pathological consistency (46, 61). Therefore, a more feasible approach is not to treat any single DUB as a “universal biomarker,” but rather to integrate it into a stratification system comprising APC/TP53, MSS/MSI, immune cell infiltration, and treatment history (4, 46) (**Table 3**).

**Table 3:** Summary of representative DUBs, evidence maturity, and biomarker potential across different cancer biomarker scenarios in the CRC.

Key CRC phenotypes	Representative DUB	Evidence maturity	Key substrates/pathways	Potential value as a biomarker	Conversion tip
DDR/chemotherapy resistance	USP1; USP10; USP28 (9, 20–22, 62) (emerging)	USP 1/USP 10 is stronger; USP 28 is weaker	FANCD2-PCNA; XAB2(K593)-ANXA2	Predicting sensitivity to platinum-based/PARP combination therapy	Priority could be given to stratification based on oxaliplatin resistance and DNA repair dependency
Differentiation/Wnt/EMT	USP7; USP10; USP44 (5, 6, 19, 28)	Moderate to strong, and background-dependent	β-catenin; Axin1; DDX3X	Note: Vulnerability in APC’s truncated background	This is better suited for interpretation in conjunction with the APC/TP53 genotype
Metabolic reprogramming	OTUB2; USP28; USP14 (12, 22, 31–33)	OTUB2/USP14 is strong; the rest are moderate	PKM2; FOXC1; IDO1	Indicates glycolysis and immunometabolic suppression	Should be evaluated in conjunction with lactate levels and immune infiltration
Iron-mediated cell death/apoptosis threshold	USP11; OTUB1; USP5 (33–35)	Intermediate	LSH/CYP24A1; GPX4; SLC7A11; Sirtuin 1(SIRT1)/GSDME	Indicates sensitivity to cell death and response to treatment	May be used in combination with chemotherapy/radiotherapy or ferroptosis-inducing strategies
Immune Checkpoints and IFN Responses	USP22; USP7; USP4 (7, 42–44)	stronger	PD-L1; TRAF6/IRF3; Antigen presentation	Predicting ICI response and MSS cold tumor status	Suitable for use in combination with PD-L1, IFN response scores, and MHC-I to construct composite indices
Myelosuppression	USP15 (46)	Intermediate	SMYD3/CCL2	Tip for MDSC	More suitable as a tool for combination therapy and companion diagnostics

## 8 Discussion

### 8.1 Comparison of CRC-specific and pan-cancer DUB regulation

Although various DUBs are commonly involved in DNA damage repair, metabolic reprogramming, immune evasion, and treatment resistance across different cancer types, their functions in CRC are not entirely identical. When writing a review, one of the most critical pitfalls to avoid is equating “has been reported in CRC” directly with “CRC-specific regulation.” These two concepts are not synonymous. The former merely indicates that evidence for a particular molecular axis exists in CRC; the latter implies that the mechanism exhibits a clear tumor-type bias, depends on a CRC-specific molecular context, or at least has substantial support from cross-cancer comparisons. Based on this, this paper tends to classify existing DUB mechanisms into three categories: (1) regulatory pathways that are clearly dependent on specific molecular backgrounds in CRC; (2) pathways that have been validated in CRC but are essentially more likely to be shared across multiple cancer types; and (3) mechanisms currently inferred primarily from non-CRC studies that require further validation in CRC models (2, 4). The significance of this classification lies not only in conceptually distinguishing between mechanisms reported in CRC and those dependent on a CRC background, but also in providing a clearer basis for subsequent stratified research and clinical translation decisions.

Based on current evidence, the examples most closely associated with CRC-context-dependent mechanisms are primarily concentrated in the context of *APC* truncation/high Wnt activation and TP53 mutation (2, 6, 30). The former is significant because APC inactivation and the resulting abnormal Wnt/β-catenin activation constitute one of the most representative molecular foundations of CRC. In this context, USP7 mediates β-catenin deubiquitination and sustains abnormal Wnt activation in APC-mutated CRC. Recent studies further suggest that USP10 stabilizes β-catenin, maintains tumor stemness, and supports the growth of patient-derived organoids in APC-truncated CRC. It should be noted that Wnt/β-catenin-related protein homeostasis regulation in CRC is not limited to DUBs; other UPS components can also participate in the regulation of this pathway by influencing the stability of key cofactors (63). Therefore, it is more accurate to say that “Wnt-related regulation is not inherently a DUB-specific advantage,” but rather that CRC presents a highly protein-homeostasis-sensitive APC/Wnt context, and certain DUBs happen to occupy key nodes within this context.

Similarly, USP7 in the context of TP53 mutations is more closely associated with “CRC-context-dependent” regulation rather than a simple, replicable pathway template across cancers. In TP53-mutated CRC, USP7 can directly stabilize mutant p53 and is associated with cancer stem cell maintenance and tumor growth (5). What truly merits emphasis here is not that “USP7 is associated with p53 in many cancer types,” but rather that a significant proportion of CRC cases involve TP53 abnormalities, causing USP7 to exhibit distinctly different biological effects under different genotypes. It is precisely for this reason that USP7 in CRC is better understood as a “functional node highly dependent on molecular context” rather than being classified as a “universal oncogenic DUB”.

In contrast, although pathways such as USP1–FANCD2/PCNA, USP14–IDO1 or JNK, and USP22–PD-L1 have been validated in CRC, their biological themes are more likely to represent pan-cancer shared mechanisms (9, 12, 42). They certainly hold significance for CRC, but this significance stems more from the fact that “this pathway is also active in CRC” rather than “this pathway is active exclusively in CRC”.

Based on the current literature, the overall conclusion of this paper is that the “specificity” of DUB regulation in CRC is more a reflection of dependence on specific contexts rather than “tumor-type exclusivity” in an absolute sense. The priority for future research should not be to continue expanding the list of “CRC-specific DUBs,” but rather to combine CRISPR-mediated disruption screens, organoid models, and patient-derived systems to truly distinguish which DUBs are merely observed in CRC and which are critical nodes that are genuinely sensitive to the unique molecular landscape of CRC.

### 8.2 Translational challenges and clinical feasibility of targeting DUBs

Although DUBs are widely regarded as potential drug targets, their clinical translation still faces multiple obstacles, and these obstacles often prove more complex in CRC than in *in vitro* models. The first challenge is selectivity. Most DUBs, particularly those in the USP family, have highly conserved catalytic domains; consequently, active site inhibitors are prone to intra-family cross-inhibition and off-target effects (4, 8, 51–54). This is particularly critical for CRC, as targets such as USP7, USP10, and USP22 inherently exhibit significant context-dependence: the same inhibitory effect may lead to different or even opposite phenotypic outcomes depending on the specific *APC, TP53*, or immune context (5, 6, 12, 30). In other words, the difficulty in targeting DUBs in CRC lies not merely in whether a drug is “selective enough,” but in the fact that even with sufficient selectivity, consistent biological outcomes may not be achieved across all patients.

The second obstacle is the therapeutic window and toxicity. DUBs are extensively involved in protein homeostasis, DNA repair, cell cycle regulation, and immune homeostasis; true target-related toxicity is often difficult to completely (4, 52). Take USP1 as an example: while its inhibition holds promise for enhancing the therapeutic response to DNA damage, it is also necessary to consider the dependence of normally proliferating tissues on FANCD2-mediated DNA repair. Since USP1 is a key deubiquitinating enzyme in the Fanconi anemia/FANCD2 axis, and this pathway is closely linked to hematopoietic stem/progenitor cell homeostasis and bone marrow failure phenotypes, the potential risks of bone marrow suppression or impaired DNA repair in normal tissues must be evaluated in advance when advancing USP1 inhibition in CRC; its translational value cannot be judged solely based on tumor cell sensitivity (4, 9, 64). Similarly, although USP7 inhibition holds strong biological appeal, its broad substrate profile and significant context dependency make both efficacy and adverse effects more difficult to predict (7, 8).

The third obstacle is compensatory upregulation and network rewiring. The DUB network inherently possesses a degree of redundancy; when one target is inhibited, other DUBs may exert compensatory effects on certain substrates. Additionally, CRC itself exhibits strong pathway plasticity and therapeutic adaptability, implying that inhibiting a single DUB is likely to disrupt the system only temporarily, rather than achieving sustained suppression (2, 4, 52). Existing studies even suggest that compensatory changes in bypass DUBs may occur following USP7 inhibition: for example, FT671 and other USP7 inhibitors can induce USP22 upregulation in cells such as HCT116, suggesting that the system may restore partial deubiquitination capacity through rewiring following a single DUB intervention (56, 57, 65). Therefore, based on the current literature, a more realistic approach to DUB targeting in CRC is not to rely solely on long-term monotherapy, but rather to develop interpretable combination strategies with chemotherapy, DNA damage therapy, immunotherapy, or metabolic interventions (66).

The fourth issue concerns PK/PD and delivery. Traditional small molecules are often limited by insufficient *in vivo* exposure, limited tissue penetration, and the absence of pharmacodynamic biomarkers; while PROTACs and DUBTACs, although offering new functional coverage, simultaneously introduce issues such as higher molecular weight, poor cellular permeability, limited tissue distribution, and more complex pharmacodynamic assessment. For CRC, these limitations are further amplified by the intestinal environment, intratumoral heterogeneity, and differences in the immune microenvironment (4, 59, 60). Therefore, what truly determines whether a DUB approach can advance to clinical trials is often not its *in vitro* efficacy, but rather its ability to maintain achievable exposure, traceable efficacy, and acceptable safety margins within the solid tumor environment (59, 67).

From a cross-cancer comparison perspective, DUB research in fields such as breast cancer, pancreatic cancer, and glioma typically enters the discussion phase—centered on pharmacokinetic optimization, model stratification, and patient stratification—earlier than in CRC. For CRC, the significance of this comparison lies not in directly adopting target lists from other cancer types, but in drawing on their development logic: First, clarify the most critical biological context; then define the patient populations likely to benefit; and subsequently advance pharmacodynamic optimization and combination strategy design (4, 8, 52). In other words, what CRC currently lacks most is not “the next new DUB,” but rather the evaluation of existing targets within a clinical translation framework.

Taken together, it is the combination of issues such as selectivity, therapeutic window, network rewiring, and PK/PD and delivery that makes DUB-targeted therapy in CRC more suitable for context-dependent combination therapy rather than broad-spectrum monotherapy development. Based on current evidence, this paper tends to argue that a more realistic path forward for DUB-targeted therapy in CRC is not to pursue “a single DUB effective against all CRC cases,” but rather to establish a translational framework combining “biomarker stratification —combination therapy—dynamic efficacy monitoring (5–9). The DUB-targeted strategies that hold genuine promise for future clinical translation may not necessarily be the earliest-discovered or most potent *in vitro* molecules, but rather those that can achieve stable therapeutic effects in specific contexts, possess a logical basis for combination therapy, and can be tracked by biomarkers.

### 8.3 An integrated model of DUB-mediated CRC progression

A comprehensive review of the literature reveals that DUBs do not act in isolation in CRC. On the one hand, they are influenced by factors such as the APC/TP53 background, replicative stress, hypoxia, metabolic stress, and exposure to therapeutic agents; on the other hand, they can further modulate key phenotypes including DNA repair capacity, metabolic adaptation, sensitivity to cell death, antigen presentation efficiency, and the intensity of immune suppression (2, 7, 12, 42–44, 46). Therefore, DUBs in CRC are better understood as “bridge-like regulatory nodes” rather than as a series of independent deubiquitination events. In other words, they are not simply distributed across parallel modules such as the DDR, metabolism, cell death, and immune evasion, but rather functionally link these processes by influencing a few key thresholds.

**Figure 7:**
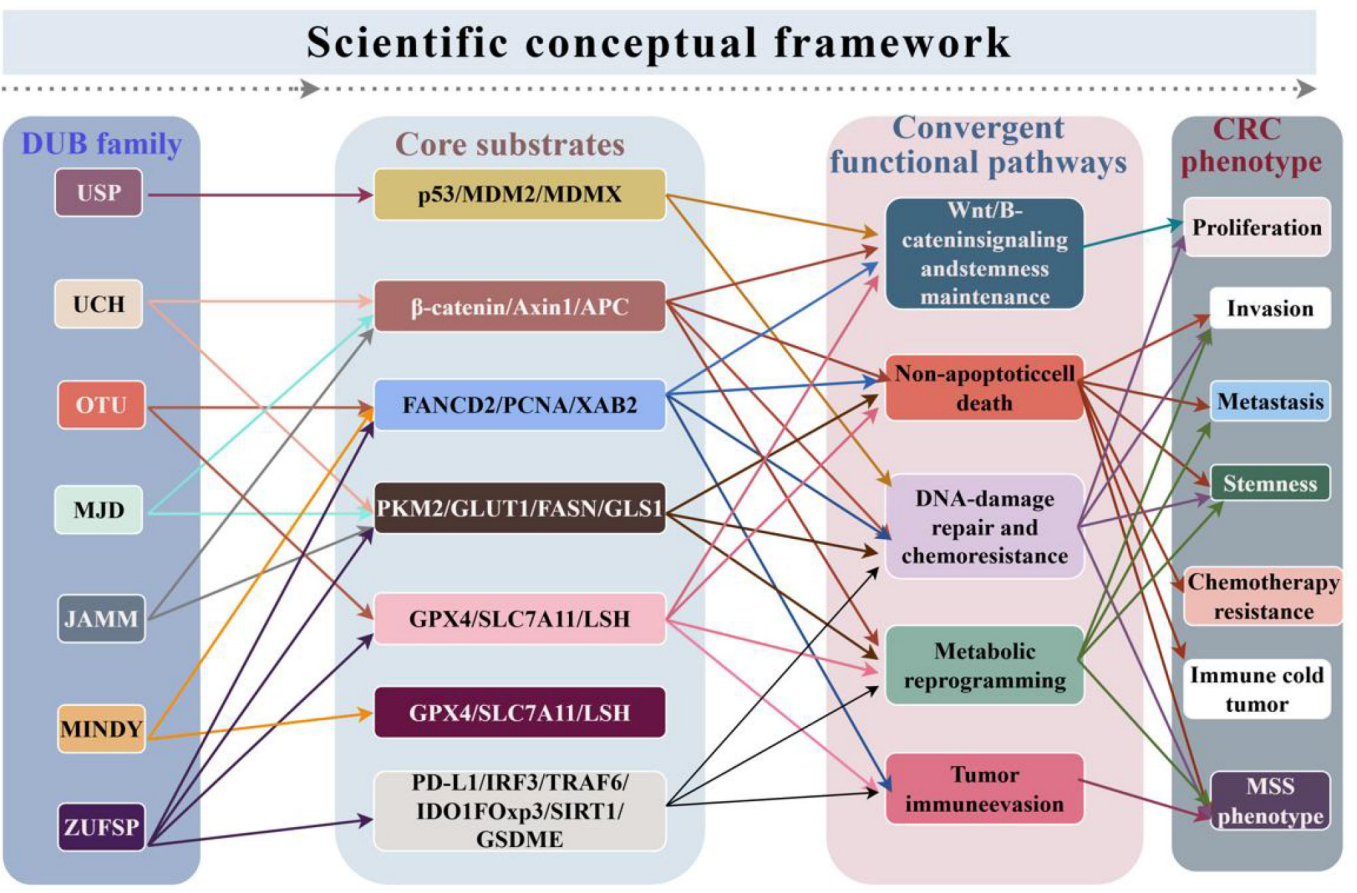
An integrated model of DUB-mediated CRC progression. The left panel shows the major contextual factors influencing DUB biological outputs, including genotypic background, microenvironmental stress, and therapeutic inputs; the middle panel depicts four major functional levels regulated by DUBs within tumor cells: DNA damage response and repair, metabolic adaptation, non-apoptotic cell death thresholds, and immune phenotype remodeling; The right panel depicts the ultimate clinically relevant outcomes, such as stemness maintenance, treatment tolerance, the MSS cold tumor phenotype, and potential windows for combination therapy (2–4). The core objective of this figure is not to mechanically summarize the mechanisms described earlier, but rather to highlight how different DUB axes functionally converge in CRC onto a few key thresholds that determine tumor progression and treatment response, thereby providing an overarching framework for subsequent stratified research and translational design.

From an integrative modeling perspective, DUB-mediated actions in CRC can be broadly categorized into three layers. The first layer is the background-dependent layer, where APC/Wnt imbalance, TP53 status, MSS/MSI immunological landscape, replicative stress, and therapeutic exposure collectively determine whether different DUB axes are activated and gain functional advantage (5, 7). The second layer is the functional execution layer, wherein DUBs shape the survival and adaptive capabilities of tumor cells by regulating processes such as the DNA damage response, metabolic reprogramming, non-apoptotic cell death thresholds, and immunological visibility (12, 46). The third layer is the phenotypic output layer, which manifests as clinically relevant outcomes such as chemotherapy resistance, maintenance of cellular plasticity, formation of an immunosuppressive microenvironment, and poor response to immunotherapy (12, 20, 46). Based on this logic, **Figure 7** is not a simple compilation of the content from the preceding sections, but rather emphasizes that although different DUB axes have different starting points, they ultimately converge on a few key thresholds that determine CRC progression and treatment response.

This integrated perspective is particularly important for understanding the true significance of DUB research in CRC. It suggests that future research should prioritize not simply adding “yet another DUB associated with CRC,” but rather identifying which DUBs truly lie at the intersection of multiple key phenotypic axes. In other words, molecules with greater potential translational value are often not those involved in a single, localized pathway, but rather bridge-type DUBs that simultaneously influence DNA damage adaptation, metabolic state, and immune visibility. Only by establishing a clearer functional hierarchy at this level can DUB-targeting strategies progress from being merely mechanistically interesting to having clinical relevance.

Based on the current literature, this paper tends to believe that the most important direction for the next phase of DUB research in CRC is not to continue expanding the list of molecules, but to further clarify, following the logic of “background—node—phenotype—translation,” which DUBs are merely local regulators and which truly determine therapeutic vulnerability and windows for combination therapy (**Figure 7**).

## 9 Limitations and future directions

Although DUB research is gradually integrating the disparate aspects of DNA damage response, metabolic reprogramming, cell death, and immune evasion within the field of CRC, the current body of evidence still has several notable limitations. First, the substrate recognition specificity and context-dependence of most DUBs have not yet been fully elucidated. For many molecules, current understanding remains limited to “upregulated expression or functional relevance,” while the structural basis of their interactions with substrate proteins, preferences for specific ubiquitin chain types, spatiotemporal dynamics, and microenvironment-dependent regulatory networks remain poorly characterized (4, 52). This explains why the same DUB may exhibit biologically divergent effects in different CRC contexts.

Second, many DUB mechanisms in CRC are currently based primarily on *in vitro* cell models or limited animal experiments, and high-level evidence remains insufficient. Particularly in studies related to the tumor immune microenvironment, how DUBs establish cross-cell-type synergistic regulation among tumor cells, myeloid cells, and lymphocytes has not yet been systematically elucidated (46–49). This implies that while existing studies have suggested DUBs may link the intrinsic survival advantages of tumor cells to the external immunosuppressive environment, it remains necessary to clarify further which effects represent truly decisive direct mechanisms in CRC and which are primarily inferred from pan-cancer or immunological contexts.

Third, at the clinical translation level, DUB research still faces several practical obstacles. Given the high conservation of DUB catalytic domains, significant tumor heterogeneity, and the lack of stable and reliable predictive biomarkers, the development of highly selective inhibitors is challenging. Additionally, single-target interventions may lead to compensatory upregulation, network rewiring, and acquired resistance, making “long-term suppression with a single agent” an unrealistic development pathway in CRC (4, 8, 51–54). As certain DUB inhibitors gradually enter clinical exploration, potential resistance mechanisms must also be addressed proactively, such as compensatory takeover by functionally similar DUBs, weakened drug binding due to alterations in the target or associated proteins, and feedback activation of bypass signaling pathways such as autophagy and PI3K/AKT. A forward-looking understanding of these issues will directly influence the rational design of subsequent combination therapy regimens.

Given these limitations, future research should prioritize at least four key directions. First, by integrating CRISPR/Cas9 functional screening, we should identify the critical DUB nodes that truly determine treatment susceptibility in models such as oxaliplatin resistance, radiotherapy response, and immune evasion, rather than continuing to expand the list of molecules in parallel. Second, quantitative proteomics and ubiquitinomics should be combined with functional disruption to distinguish between the two distinct states of “upregulated expression” and “enhanced ubiquitin chain editing activity,” thereby improving mechanistic understanding. Third, single-cell multi-omics, spatial transcriptomics, organoids, and patient-derived xenograft (PDX) models should be further integrated to more accurately define the functional boundaries of DUBs across different cell types, particularly their hierarchical functions among tumor cells, myeloid cells, and lymphocytes. Fourth, greater emphasis should be placed on the systematic validation of synthetic lethality and combination therapy strategies, such as the combination of DDR-related DUB inhibition with PARP/platinum-based therapy, or the combination of immune-related DUB intervention with PD-1/PD-L1 blockade (4, 7, 9, 12, 44, 46, 52).

From the perspective of this paper, the practical value of DUBs in CRC is, in the short term, better suited as a key entry point for mechanistic research and the design of combination therapies, rather than being viewed as a universal monotherapy target. What will truly drive progress in this field in the future may not depend on the discovery of additional new DUBs, but rather on the ability to establish an integrated “background-node-phenotype-translation” research framework centered on patient stratification, efficacy biomarkers, drug selectivity, and combination strategies. Only within this framework is DUB research more likely to transition from the current stage of accumulating mechanistic knowledge to a translational phase that genuinely contributes to precision therapy for CRC.

## 10 Conclusion

Overall, the role of DUBs in CRC is not a simple sum of disparate molecular mechanisms, but rather a network of protein homeostasis regulation that spans multiple critical levels, including the DNA damage response, metabolic reprogramming, non-apoptotic cell death, and immune evasion. Although DUBs from different families differ in their substrate profiles and modes of action, their functions in CRC often converge on a few key thresholds that determine tumor progression and treatment response, including adaptation to replicative stress, maintenance of cellular plasticity, reduced immunodetectability, and the development of treatment resistance. Therefore, they are better understood as bridge-like regulatory nodes connecting the molecular background, tumor phenotype, and therapeutic strategies, rather than as isolated deubiquitination events. Based on current evidence, the maturity of DUB-related mechanisms in CRC varies: relatively more direct evidence is primarily concentrated in the USP1, USP7, USP22, USP44, OTUB2, USP14, USP11, OTUB1, USP5, USP4, and USP15, whereas copper-mediated cell death, certain forms of pyroptosis, and several intrinsic mechanisms of immune cells currently rely more heavily on insights from pan-cancer or immunological studies. Based on the existing literature, the “specificity” of DUB regulation in CRC is more a reflection of dependence on specific contexts rather than an absolute tumor-type exclusivity. Therefore, future research should not merely focus on expanding the list of “CRC-associated DUBs,” but rather on further identifying which DUBs truly determine treatment sensitivity, windows for combination therapy, and immunoresponsiveness. At the clinical translation level, DUB research is currently better suited as a key entry point for mechanistic studies and combination therapy design, rather than simply serving as a universal monotherapy target. Although strategies such as small-molecule inhibitors, PROTACs, and DUBTACs are gradually expanding the druggability landscape of DUBs in CRC, their clinical advancement remains constrained by factors such as context dependency, selectivity, toxicity windows, compensatory rewiring, and the lack of efficacy biomarkers. In the future, if an integrated research pathway encompassing “background—node—phenotype—translation” can be further established around patient stratification, efficacy biomarkers, drug selectivity, and combination strategies, DUB research is expected to provide more interpretable and practically meaningful insights for elucidating the mechanisms of CRC and advancing precision therapy.
